# Detecting differential alternative splicing events in scRNA-seq with or without Unique Molecular Identifiers

**DOI:** 10.1371/journal.pcbi.1007925

**Published:** 2020-06-05

**Authors:** Yu Hu, Kai Wang, Mingyao Li

**Affiliations:** 1 Department of Biostatistics, Epidemiology and Informatics, University of Pennsylvania Perelman School of Medicine, Philadelphia, Pennsylvania, United States of America; 2 Center for Cellular and Molecular Therapeutics, Children’s Hospital of Philadelphia, Pennsylvania, United States of America; 3 Department of Pathology and Laboratory Medicine, University of Pennsylvania, Philadelphia, Pennsylvania, United States of America; Carnegie Mellon University, UNITED STATES

## Abstract

The emergence of single-cell RNA-seq (scRNA-seq) technology has made it possible to measure gene expression variations at cellular level. This breakthrough enables the investigation of a wider range of problems including analysis of splicing heterogeneity among individual cells. However, compared to bulk RNA-seq, scRNA-seq data are much noisier due to high technical variability and low sequencing depth. Here we propose SCATS (Single-Cell Analysis of Transcript Splicing) for differential splicing analysis in scRNA-seq, which achieves high sensitivity at low coverage by accounting for technical noise. SCATS models scRNA-seq data either with or without Unique Molecular Identifiers (UMIs). For non-UMI data, SCATS explicitly models technical noise by accounting for capture efficiency and amplification bias through the use of external spike-ins; for UMI data, SCATS models capture efficiency and further accounts for transcriptional burstiness. A key aspect of SCATS lies in its ability to group “exons” that originate from the same isoform(s). Grouping exons is essential in splicing analysis of scRNA-seq data as it naturally aggregates spliced reads across different exons, making it possible to detect splicing events even when sequencing depth is low. To evaluate the performance of SCATS, we analyzed both simulated and real scRNA-seq datasets and compared with existing methods including Census and DEXSeq. We show that SCATS has well controlled type I error rate, and is more powerful than existing methods, especially when splicing difference is small. In contrast, Census suffers from severe type I error inflation, whereas DEXSeq is more conservative. When applied to mouse brain scRNA-seq datasets, SCATS identified more differential splicing events with subtle difference across cell types compared to Census and DEXSeq. With the increasing adoption of scRNA-seq, we believe SCATS will be well-suited for various splicing studies. The implementation of SCATS can be downloaded from https://github.com/huyustats/SCATS.

## Author summary

Alternative splicing is a major mechanism for generating transcriptome diversity. However, few published scRNA-seq studies have investigated alternative splicing, and even when studied, methods developed for bulk RNA-seq were utilized. Compared to bulk RNA-seq, scRNA-seq data are much noisier due to high technical variability and low sequencing depth. Methods developed for bulk RNA-seq may not be optimal when analyzing data generated from scRNA-seq experiments. To fill in this gap, we developed SCATS, an open-source software package, which allows analysis of scRNA-seq data with or without Unique Molecular Identifiers (UMIs). SCATS is able to detect splicing events even when sequencing depth is low. When applied to mouse brain scRNA-seq datasets, SCATS identified more differential splicing events with subtle differences across cortical cell types than Census and DEXSeq. Additionally, SCATS accurately characterized splicing heterogeneity across cortical cell types, which was further confirmed by qRT-PCR measurements. Our study highlights the benefit of SCATS for elucidating splicing heterogeneity across cells in scRNA-seq data.

## Introduction

The emergence of scRNA-seq technology has made it possible to measure gene expression variations at cellular level. This breakthrough enables the investigation of a wide range of problems including analysis of splicing heterogeneity among individual cells. However, compared to bulk RNA-seq, scRNA-seq data are much noisier due to high technical variability, low sequencing depth, and the lack of full-length transcript sequencing for droplet-based protocols. Despite the growing popularity of scRNA-seq, few published studies have investigated alternative splicing, and even when studied, methods developed for bulk RNA-seq were utilized [1–3], which may not be optimal for scRNA-seq data.

Methods designed specifically for splicing analysis in non-UMI based scRNA-seq data only started to emerge recently [4]. Huang *et al*. [5] detects differential exon-usage by performing a pairwise comparison between every two cells, which becomes computationally infeasible when large number of cells are generated from droplet-based protocols [6–8]. Song *et al*.[9] quantifies exon-inclusion levels based on junction-spanning reads for non-UMI based scRNA-seq data. However, these estimations are unreliable due to sparse read counts that span exon-exon junctions and technical noise of scRNA-seq data, leading to limited power in detecting DAS events. Qiu *et al*. [10] and Ntranos *et al*. [11] developed approaches to detect differential transcript usage based on pre-estimated cell-specific isoform expressions or transcript compatibility counts. Although encouraging, the feasibility of estimating isoform usage at single-cell level still remains questionable due to limited informative reads for splicing in scRNA-seq.

**Fig 1:**
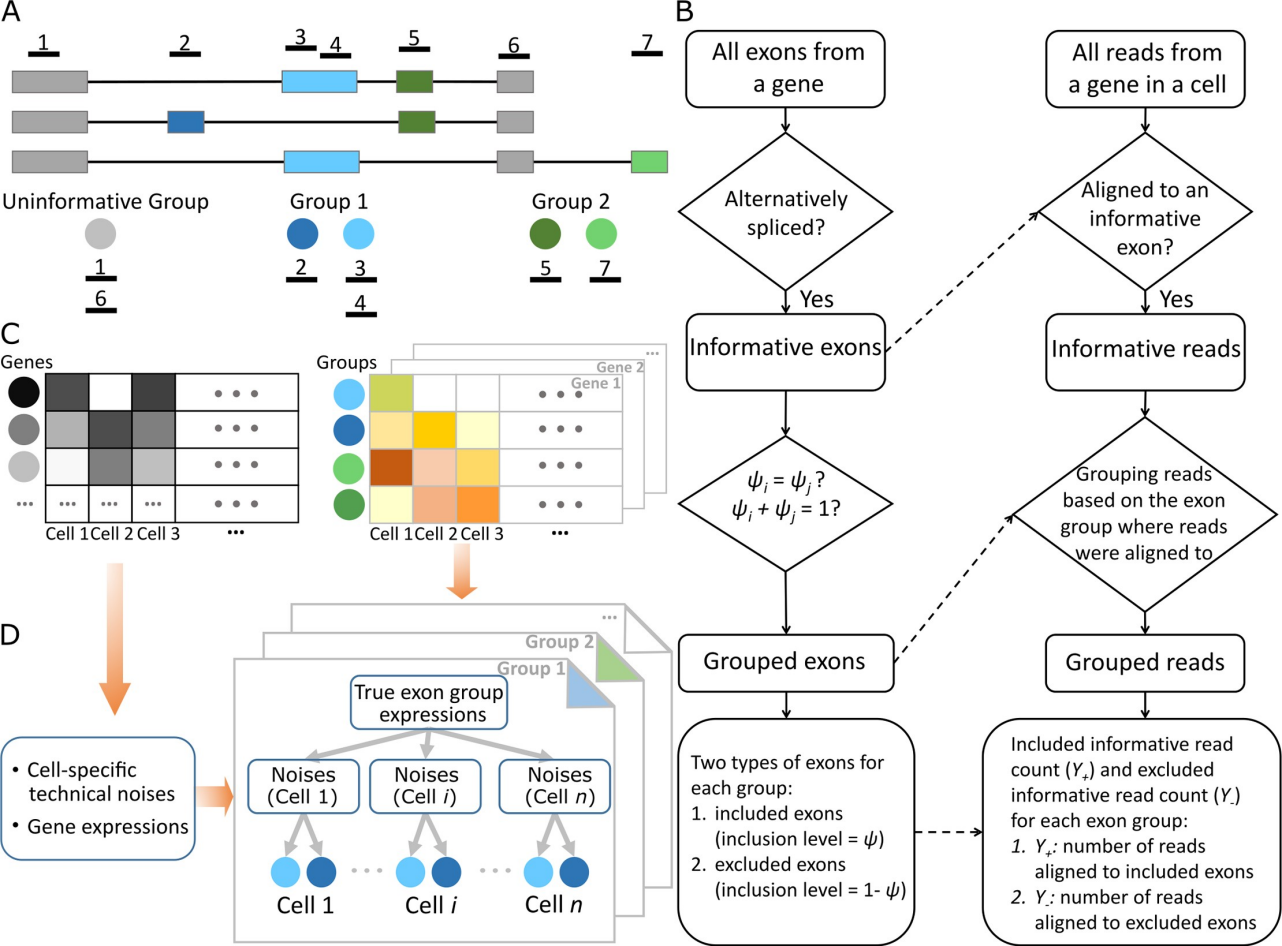
Workflow of SCATS. **(A)** Exons were first divided into groups based on their isoform origin. Those exons that originate from the same isoform(s) share the same exon-inclusion level and are thus grouped together. Group 1 (blue) and group 2 (green) are informative for alternative splicing, while uninformative group (grey) is not. Within each exon group, exons were further classified to included (inclusion level = *ψ*) and excluded (inclusion level = 1−*ψ*) region. Dark blue and dark green represent included regions. Light blue and light green represent excluded regions. **(B)** Flow chart of exon grouping and informative read grouping algorithm. **(C)** Observed read counts of scRNA-seq. Gene-level read count matrix (left) and group-level informative read count matrices (right) are summarized from aligned scRNA-seq data in BAM format. **(D)** Given gene-level read count matrix, cell-specific technical noises and population level gene expressions are quantified using TASC [17]. Given technical parameter estimates from **(C)**, for each alternative spliced exon group, the statistical inference (exon-inclusion level estimation and inclusion level difference testing) on exon-inclusion levels was made based on a hierarchical model that accounts for technical noise.

Here we present SCATS, which achieves high sensitivity to detect DAS events in scRNA-seq by accounting for technical noise and low sequencing depth through an exon-grouping approach originally developed in PennDiff [12]. Given annotated transcript information, sequencing reads aligned to exons originated from the same isoform(s) are first grouped together (**Fig 1A**). This grouping step is essential in splicing analysis of scRNA-seq data as it naturally aggregates spliced reads across different exons, making it possible to detect DAS events even for genes with low sequencing depth (**Fig 1B, Fig 1C, Fig 1D**, **S1 Fig**). Moreover, SCATS can be applied to scRNA-seq data either with or without UMIs. For non-UMI data, SCATS explicitly models technical noise by accounting for capture efficiency, amplification bias and dropout events through the use of external spike-ins; for UMI data, SCATS models capture efficiency and further accounts for transcriptional burstiness in detecting DAS events between groups of cells.

## Results

### Estimation of exon-inclusion level

Exon-inclusion level, which measures the relative usage of an exon, is a commonly used measure to quantify the process of alternative splicing. A critical step in DAS detection is to reliably estimate exon-inclusion levels for exons that originate from the same isoform(s). For an alternatively spliced exon *e*, its exon-inclusion level, *x*_*e*_, is defined as the percentage of transcripts that have exon *e* included. Mathematically, it can be written as $x_{e}=\sum_{j\in I_{e}}\theta_{j}$, where *I*_*e*_ represents the set of isoforms that have exon e included, and *θ*_*j*_ represents the relative abundance of isoform *j*. Although one can estimate the relative abundance of each inclusion isoform and then sum over the relative abundances of all inclusion isoforms to estimate *x*_*e*_, this approach is inaccurate due to the extreme sparsity of scRNA-seq data. To get a more reliable estimate of *x*_*e*_, we utilize splicing informative reads at the alternatively spliced exon *e* and those exons that are in the same exon group as exon *e*. This approach is more robust than estimating isoform relative abundances directly as it naturally aggregates splicing informative reads that originate from the same isoform(s) together. As shown in **Fig 1A**, for each gene, alternatively spliced exons are grouped if they originate from the same isoform(s) or complementary isoform(s) (i.e. exons share the same inclusion levels or complementary inclusion levels). **Fig 1B** illustrates the detailed procedure of exon grouping and the identification of informative reads for splicing.

### Evaluation on simulated data

**Fig 2:**
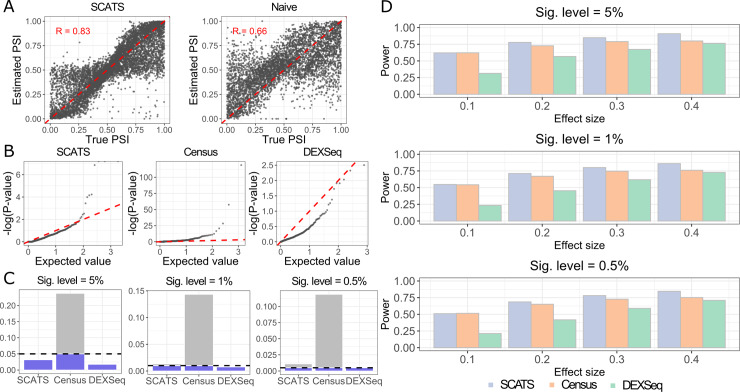
Exon-inclusion level estimation, false positive rate comparison and power comparison of simulation study. Informative read counts of 1,000 exon groups for 600 cells (300 vs 300) were simulated using a generative model shown in **S1 Fig** with cell-specific technical parameters estimated from CA1 pyramidal cells in the scRNA-seq data generated by Zeisel et al. [16]. To simulate data that resemble real scRNA-seq data, one CA1 pyramidal cell was randomly selected and read coverage for each gene in that cell was used to obtain true gene concentration at the population level ($gene\;concentration=100,000\times \frac{\#reads\;from\;the\;gene}{\#total\;number\;of\;reads\;from\;the\;CA1\;pyramidal\;cell}$). Based on the generative model accounting for technical noise, we generated 6,000 reads on average for each cell. **(A)** Scatter plots of 1,842 true exon-inclusion levels, estimated by percent spliced in (PSI) for a given exon, against estimates from SCATS and a naïve method that ignores technical noise. SCATS models technical noise to quantify usage of an alternatively spliced exon while the naïve method simply estimates the inclusion level by $\psi =\frac{Y_{+}}{Y_{+}+Y_{-}}$, where *Y*_+_ and *Y*_−_ are the informative read counts of the “+” and “-” exon groups across cells. SCATS estimates are closer to the ground truth. **(B)** Quantile-quantile plots of the p-values from SCATS, Census and DEXSeq under the null hypothesis (Δ = 0). X-axis represents uniform theoretical quantiles between 0 and 1 in –log_10_ scale. Y-axis represents observed p-value quantile in –log_10_ scale. Uniformly distributed data should follow the red dashed line. P-values of SCATS are more uniformly distributed while those from Census are right-skewed and those from DEXSeq are left-skewed. **(C)** Type I error comparison of SCATS, Census and DEXSeq with different significance levels (α = 0.05, 0.01, 0.005). Similar to **(B)**, SCATS has better type I error control than Census and DEXSeq. **(D)** Power comparison of SCATS, Census and DEXSeq. Barplots show the estimated power under different effect sizes (Δ = 0.1,0.2,0.3,0.4), where significance was evaluated at 0.05, 0.01, and 0.005 levels, respectively. Colors indicate different methods. SCATS outperformed Census and DEXSeq across all effect sizes, especially when Δ = 0.1. DEXSeq is conservative in detecting DAS events.

To evaluate the effectiveness of exon grouping for exon-inclusion level estimation, we simulated data from a generative model (see **Material and Methods** for details). Our simulations indicate that SCATS’s estimates are strongly correlated with the true levels (R = 0.83), whereas naïve estimation that ignores technical noise in scRNA-seq only leads to a correlation of 0.66 (**Fig 2A**). We further assessed the performance of SCATS in detecting DAS events, and compared with two other state-of-the-art methods: Census [10], an algorithm designed to preprocess raw read counts by removing technical noise, and DEXSeq [13], a popular method for bulk RNA-seq splicing analysis. Specifically, Census estimates isoform expression levels first and converts them into Census RNA counts, and then fits a Dirichlet multinomial mixture model to detect differential splicing events. DEXSeq identifies differential exon usage by fitting exonic counts with a log linear regression model in which exonic counts are obtained by counting the number reads aligned to a virtual exon. Our results indicate that SCATS has well controlled type I error rates (**Fig 2B, Fig 2C**), whereas Census tends to yield false positive results with enrichment of p-values near zero, and DEXSeq is overly conservative with enrichment of p-values near one. SCATS is also more powerful than Census and DEXSeq (**Fig 2D**), although Census has severely inflated type I error rates. Compared to DEXSeq, SCATS performs consistently better, especially when the true exon-inclusion level difference is small. This indicates that SCATS is robust in detecting subtle splicing difference, thanks to the accurate exon inclusion-level estimation due to exon grouping and technical noise modeling.

The above simulations generated data assuming the same model underlying SCATS. To make a fair comparison, we designed a new simulator to generate an *in silico* library for a single cell (see **Material and Methods** for details). This simulator does not make any parametric assumptions on gene expression across cells. Results for data generated from this new simulation scheme are shown in **S2 Fig**, which indicate that SCATS has well controlled type I error rates (**S2A Fig, S2B Fig**), whereas Census tends to yield false positive results with enrichment of p-values near zero, and DEXSeq is overly conservative with enrichment of p-values near one. SCATS is also more powerful than Census and DEXSeq (**S2C Fig**). These results are consistent with what we observed in the original parametric simulation scheme.

To evaluate the impact of novel isoforms, we conducted a simulation study to test the robustness of SCATS to under-annotation, that is, some of the novel isoforms are not included in the annotation. Specifically, we simulated scRNA-seq read counts based on 100% Ensembl annotated isoforms and analyzed the data with SCATS using 100%, 75% and 50% of the annotated isoforms. **S3 Fig** shows the power comparison results under different degrees of under-annotation across different significance levels and effect sizes. SCATS is robust to under-annotation in all scenarios especially when effect size was large. Compared to full annotation, when only using 50% of the annotated isoforms in analysis, the power of SCATS only decreased by 4% on average across different significance levels. These results indicate the robustness of SCATS to isoform annotation. Similarly, we evaluated the impact of exon group length on SCATS. As shown in **S4 Fig**, all different length groups have well controlled type-I error rate when effect size equals to 0 and SCATS is more powerful as exon group length increased. This matches our expectation and demonstrates the improved performance of SCATS by grouping exons to account for read sparsity in scRNA-seq.

Moreover, we conducted a pseudo-bulk simulation by pooling scRNA-seq data from 300 cells in each group to compare this pseudo-bulk approach with SCATS. Since there is only one sample in each condition in the pseudo-bulk data, we cannot perform statistical tests. Therefore, we examined differential splicing by fold change (FC). We considered two significance thresholds of average fold change for analysis based on the pseudo-bulk data: FC>1.2 and FC>1.5. For the pseudo-bulk approach, the power is calculated as the proportion of exon groups with FC>1.2 or 1.5 among exon groups with differential splicing and the type-I error is calculated as the proportion of exon groups with FC>1.2 or 1.5 among exon groups with no splicing difference. As shown in **S5 Fig**, SCATS has higher power for FC>1.5 across all scenarios, especially when effect sizes are relatively small. SCATS and FC>1.5 both have well controlled type-I error rate. When effect size is greater than 0.2, FC>1.2 is more powerful than SCATS. However, FC>1.2 has severely inflated type-I error rate. These results demonstrate the benefit of SCATS over pseudo-bulk approach in detecting inclusion-level difference of scRNA-seq data.

### Application to Tasic *et al*. data

**Fig 3:**
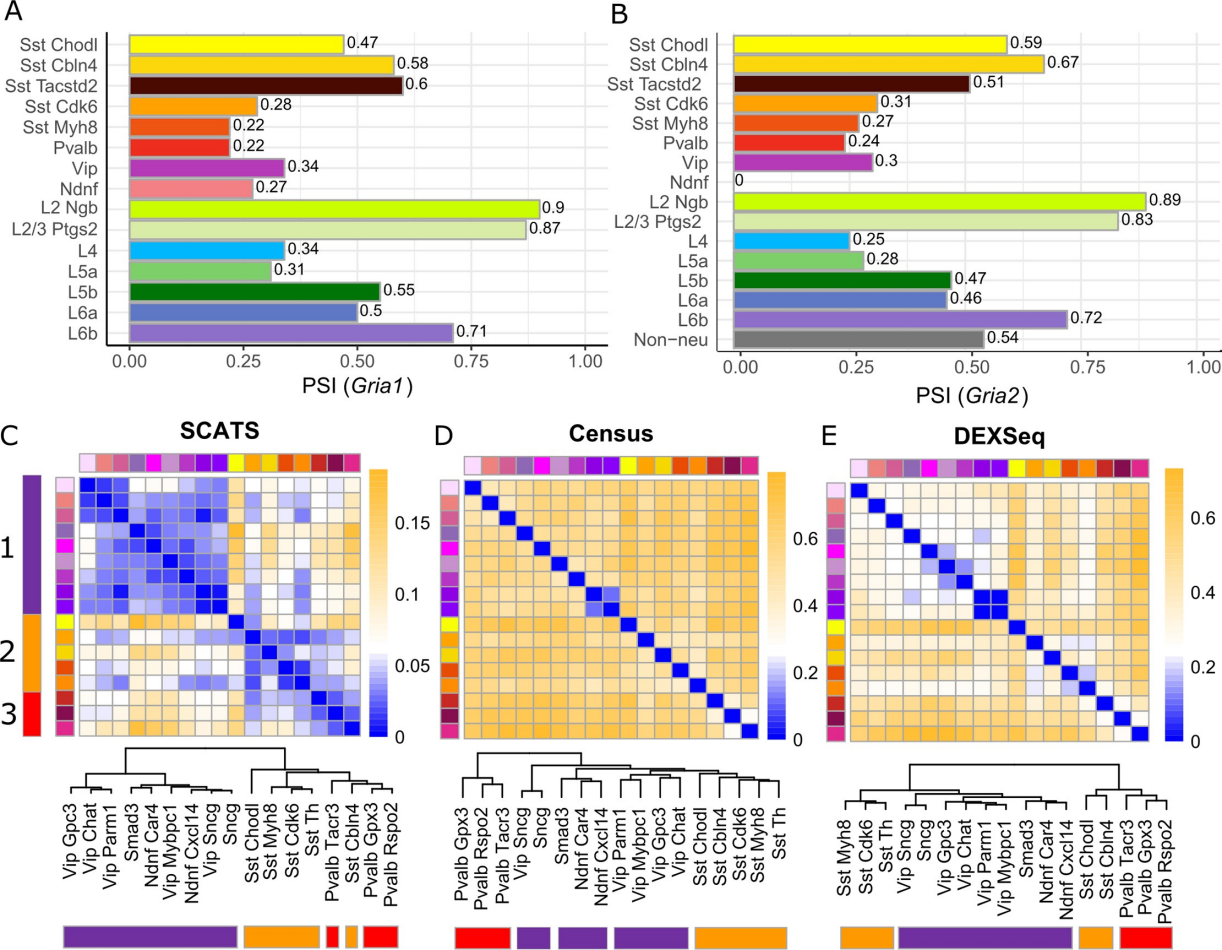
DAS analysis results of the Tasic *et al*. data. **(A,B)** Exon-inclusion level quantification of flip and flop exons from gene *Gria1* and *Gria2* using SCATS. Barplots show exon-inclusion level estimates of the flip exon from gene *Gria1* **(A)** and *Gria2* **(B)** across different cell types. Colors indicate different cell types. These estimates agree well with low-resolution RNA ISH data. **(C-E)** Pairwise DAS comparison across GABAergic cell types from mouse cortex using SCATS **(C)**, Census **(D)** and DEXSeq **(E)**. Colors indicate different GABAergic cell types, and purple, yellow, and red indicate three sub cell classes. Heatmaps show the proportion of detected DAS exon groups (SCATS) or DAS exons (Census and DEXSeq) for each pairwise comparison between cell types based on 296 genes identified by Tasic *et al*. using MISO. Dendrograms show cell classification results of the 17 GABAergic cell types. The distance metric between two cell types is the proportion of detected DAS exon groups by SCATS **(A)**, DAS exons by Census **(B)** and DEXSeq **(C)**. SCATS outperformed Census and DEXSeq in detecting splicing heterogeneity across different cell types, while controlling false positive rate.

Next, we applied SCATS to a scRNA-seq dataset generated from adult mouse brains, which includes 1,679 cells generated using the SMART-seq protocol (Tasic *et al*. dataset [3]) (see details in **Methods**). This dataset includes three major cell classes and 49 sub-cell types (23 GABAergic cell types, 19 glutamatergic cell types, and seven non-neuronal cell types). Major cell classes and sub cell types reported in the original publication were treated as ground truth to evaluate SCATS in characterizing splicing heterogeneity across cells. This dataset is non-UMI based but read counts from 92 ERCC spike-ins are available, which allow us to quantify technical variations. To assess the accuracy of exon-inclusion level estimation of SCATS, we selected two consecutive alternatively spliced exons (flip-flop exons [14]) from two Glutamate receptor genes *Gria1* and *Gria2* (**S6A Fig, S6B Fig**), which have been reported to display a highly variable splicing pattern across cell types [3, 14]. These flip-flop exons are complementary with each other, and each single one is included in a different mature RNA. Sommer *et al*. [14] measured the expression levels of the flip and flop exons, respectively, with RNA *in situ* hybridization (ISH). Their findings showed that the flip exon was preferentially utilized in layer II/III of neocortex as compared to the flop exon, whereas both exons were equally expressed in layer VI of neocortex. To evaluate the exon usage quantification of SCATS, we estimated cell type-specific exon-inclusion level of the flip exon. As shown in **Fig 3A** and **Fig 3B**, the estimated exon-inclusion levels of the flip exon in cell types L2-Ngb (layer II) and L2/3-Ptgs2 (layer II/III) are over 80%, whereas the estimates are around 50% in cell type L6a (layer VI) (*Gria1*: ${\hat{\psi }}_{flip}=0.5$ and *Gria2*: ${\hat{\psi }}_{flip}=0.46$). These results are consistent with RNA ISH measurements in Sommer *et al*. [14].

Encouraged by the accurate exon-inclusion estimates, we next conducted a pairwise DAS analysis across all 49 sub-cell types based on 296 most differentially spliced genes (with 966 exon groups) selected by Tasic *et al*. using MISO [15], a popular method developed for splicing analysis in bulk RNA-seq data. **S7 Fig** shows the quantile-quantile (QQ) plots of the log-transformed p-values from pairwise comparisons of different sub-cell types, which include sub-cell type comparisons both within major cell classes and cross major cell classes. For tests within GABAergic, glutamatergic and non-neurons, the p-values from SCATS are distributed slightly below the red diagonal line until when the significance level *α* reaches to 0.01. This pattern is consistent with the QQ plot shown in the simulations **(Fig 2B)**. In contrast, there is a sharp deviation from the diagonal line at *α* = 10^−1.5^ for all three cross-major cell class subsets, indicating that more DAS events were detected at less stringent significance thresholds. This is not surprising because more DAS exons are expected to be detected between sub-cell types from different major cell classes compared to sub-cell types that originate from the same major cell class. **S8A Fig**, **S8B Fig** and **S12 Fig** show DAS heterogeneity revealed by SCATS across the 49 sub-cell types based on the 966 exon groups in these 296 genes. The heatmap shows three clusters, consistent with the three labeled major cell classes: GABAergic, glutamatergic, and non-neuronal. Although this pattern was also revealed by MISO analysis in Tasic *et al*., SCATS results revealed more heterogeneous splicing patterns within each major cell class than MISO, demonstrating its higher sensitivity in detecting splicing heterogeneity.

For GABAergic cells, Tasic *et al*. conducted a hierarchical clustering analysis based on qRT-PCR measurements obtained from 79 marker genes for GABAergic sub-cell types. These cells were classified into three sub-clusters. To further assess the accuracy of SCATS in profiling splicing heterogeneity, we treated these qRT-PCR based clustering result as ground truth and asked whether SCATS can reveal this sub-clustering structure from its detected DAS events. MISO results in Tasic *et al*. showed homogenous DAS pattern across GABAergic cells with no cell subpopulations being clustered together. Heatmaps in **Fig 3C**, **Fig 3D** and **Fig 3E** show DAS analysis results of the 480 GABAergic cells using SCATS, Census and DEXSeq, respectively. The three sub-clusters (purple, orange, red) were identified by SCATS but missed by Census and DEXSeq. Specifically, SCATS clearly revealed different cell types in the heatmap (**S8A Fig**). However, Census suffers from severely inflated type I error rate in which most of the pairwise DAS proportions are greater than 40% in **S8C Fig**, which agrees with simulation results shown in **Fig 2**. Moreover, the dendrogram of SCATS shows that the purple cell types were separated from yellow and red cell types at the first level of the hierarchical clustering tree, whereas the yellow and red cell types were grouped together at the first level of the tree, indicating that the yellow and red cell types are more similar to each other than to the purple cell types. This pattern is consistent with the hierarchical clustering analysis based on the qRT-PCR measurements by Tasic *et al*. These results demonstrate the high sensitivity of SCATS in detecting DAS among closely related cells from the same major cell class. Additionally, we generated a sashimi plot using IGV to validate the detected DAS exons by SCATS. **S9 Fig** shows the empirical evidence of a DAS event at the flip-flop exons between Sst-Cdk6 and Sst-Cbln4 for gene *Gria1*, which is consistent with SCATS (*P* = 0.018).

We also analyzed a larger dataset that includes 6,275 genes with 12,073 exon groups that met the analysis criteria. The patterns are overall similar to what we observed for the 296 most differentially spliced genes selected by Tasic *et al*. using MISO (**S10 Fig**).

### Application to Zeisel *et al*. data

**Fig 4:**
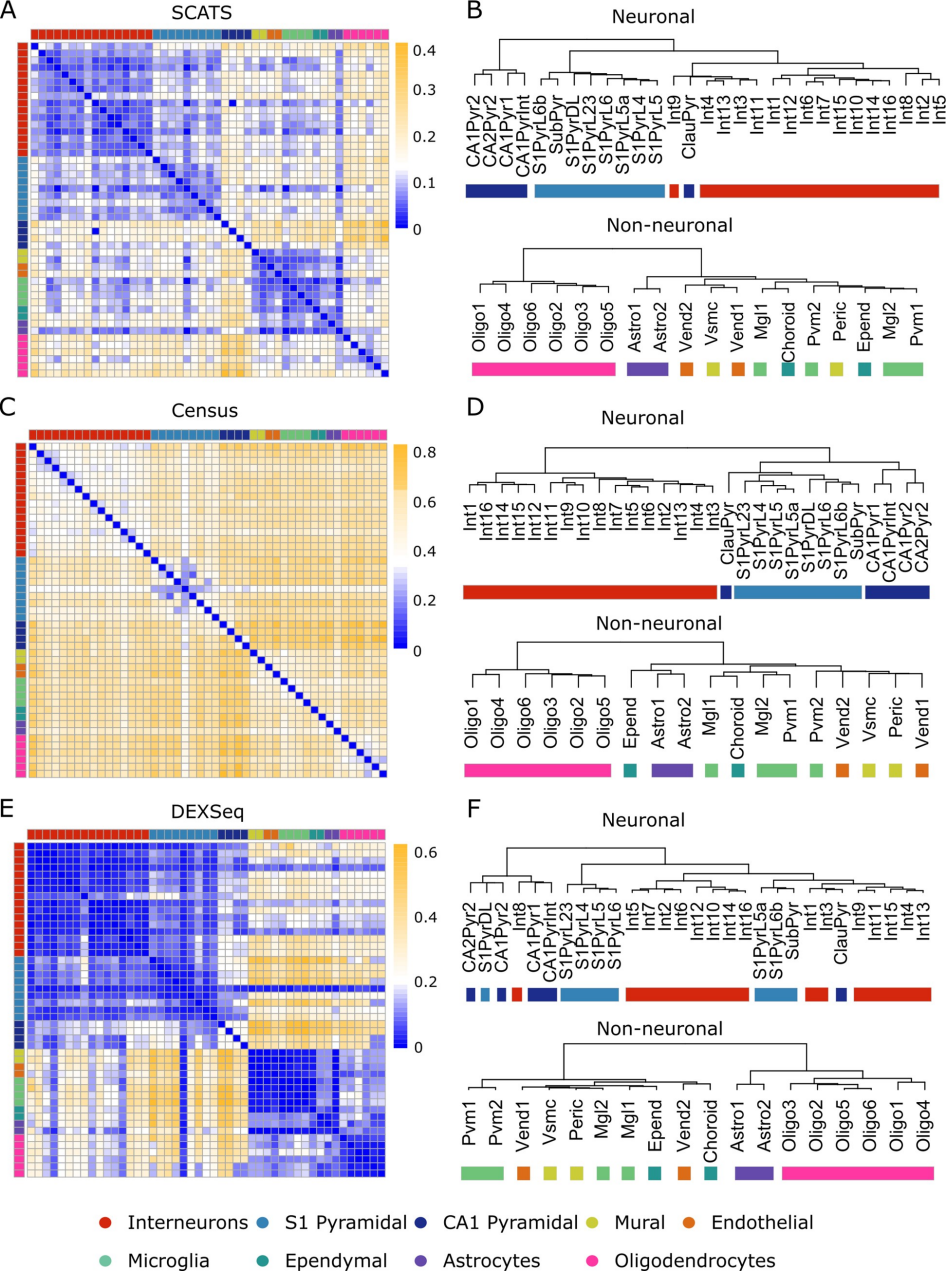
DAS analysis results of the Zeisel *et al*. data. **(A-F)** Pairwise DAS comparison across 3,005 cells (nine major cell types) from mouse cortex and hippocampus using SCATS **(A,B)**, Census **(C,D)** and DEXSeq **(E,F)**. Colors indicate nine major cell types. **(A,C,E)** Heatmaps showing the proportion of detected DAS events from 1,826 genes for each pairwise comparison between cell types. **(B,D,F)** Dendrograms showing cell classification results of the 47 sub cell types. The distance metric between two cell types is the proportion of detected DAS exons groups for SCATS **(B)**, and DAS exons for Census **(D)** and DEXSeq **(F)**. This dataset shows highly cell type-specific alternative splicing pattern.

Although designed primarily to detect DAS events in full-length transcript scRNA-seq data, SCATS can also detect DAS events for UMI data, which typically only contain sequences from the 3’ or 5’ end of a transcript. Due to the sparsity of splicing informative UMI counts, few methods are able to detect DAS events in UMI-based scRNA-seq data. To evaluate the performance of SCATS when full-length transcript information is not available, we analyzed the Zeisel *et al*. dataset [16], which are UMI-based data generated from 3,005 cells in mouse cortex. This dataset includes nine major cell classes and 47 sub-cell types. We performed DAS analysis on 1,826 marker genes (3,542 exon groups) for these nine major cell classes across the 47 sub-cell types in this dataset. Since UMI counts were consolidated from non-UMI reads, we compared read count distributions between UMI and non-UMI data. Not surprisingly, splicing informative read counts in UMI data are much sparser than non-UMI data (**S11A Fig**, **S11B Fig**), making it challenging to detect DAS in UMI data. However, by exon grouping and appropriate statistical modeling, SCATS is able to detect DAS even with such sparse data. **Fig 4A** (**S13 Fig**) shows the heatmap of the proportions of the detected DAS events for each pairwise comparison by SCATS. Neuronal cells are clearly differentiated from non-neuronal cells, although some major cell classes (marked by single colors) cannot be discerned from this heatmap. To better understand these results, we treated the proportions of significant DAS exons for each pairwise comparison as a similarity metric and performed hierarchical clustering analysis as shown in **Fig 4B**. For SCATS, the separation in neuron cells agrees well with labeled cell types, with only one CA1 Pyramidal cell type (ClauPyr) misclassified as Interneurons. For non-neuronal cells, SCATS clearly separated the Oligodendrocytes and Astrocytes cell classes from others, with four labels in Ependyma, Microglia and Mural misclassified. These clustering results demonstrate the reliability of DAS detections across cell types using SCATS even when splicing informative read counts are sparse. We also conducted similar analyses using MISO, Census and DEXSeq. Census may yield more false positive results because it detected more than 40% DAS events across most pairwise comparisons, even for those comparisons within major cell classes (**Fig 4C**, **Fig 4D**). In contrast, DEXSeq and MISO missed most of the DAS events and the hierarchical clustering analysis failed to separate different major cell classes for both neurons and non-neurons (**Fig 4E**, **Fig 4F**, **S11C Fig**, **S11D Fig**). This is not surprising as splicing informative molecule counts in UMI-based scRNA-seq data are extremely sparse, and methods developed for bulk RNA-seq data are not optimal when analyzing such data. It is worth noting that splicing quantification offers higher resolution of cellular heterogeneity than total gene expression. As shown in **S11E Fig**, exon-inclusion levels showed more distinct patterns across cell types for gene *Gria1* than total gene expression.

## Discussion

Detection of mis-regulated alternative splicing events is a critical step towards the understanding of association between isoform variations and disease. scRNA-seq data offer new insights into alternative splicing at cellular level, which helps us to understand cellular heterogeneity with higher resolution as compared to bulk RNA-seq. However, low sequencing depth and technical noise have precluded the investigation of splicing heterogeneity in most scRNA-seq studies. Several approaches have been developed to detect differential alternative splicing for scRNA-seq data. However, these methods either ignore technical noise or perform statistical tests based on unreliable isoform/exon usage estimates. To fill in such knowledge gap, we developed SCATS, which utilizes informative read counts at exon group level to achieve high sensitivity in DAS detection. By grouping exons that originate from the same isoform(s) and modeling of technical noise, SCATS aggregates spliced reads across different exons, making it possible to detect splicing events in scRNA-seq. Through simulations, we have shown that SCATS yields more accurate quantification of exon-inclusion levels than a method that utilizes empirical junction reads directly and is more powerful in detecting DAS events compared to existing methods.

Accurate quantification of exon-inclusion levels is critical for DAS detection. To estimate exon-inclusion levels more accurately, SCATS corrects local heterogeneity of read coverage to improve DAS detection accuracy. In particular, 3’ bias of UMI-based scRNA-seq data leads to most reads being distributed to the 3’ end of an isoform, which complicates exon-inclusion level quantification and detection of DAS events for exons that are far away from the 3’ end. SCATS models this non-uniform read distribution in a non-parametric manner, which upweights 3’ end in the model to remove 3’ sequencing bias of UMI-based scRNA-seq data. This bias correction together with exon grouping in SCATS makes it possible to detect DAS events at the 5’ end even when few reads are mapped to that region.

Although SCATS showed promising performance compared to existing methods, we note that SCATS cannot be applied to estimate isoform expression at cellular level because most next-generation sequencing (NGS) reads are too short to capture unique sequences for each isoform. In a typical scRNA-seq dataset generated using NGS, the number of splicing informative reads in each cell is extremely small, and in many situations is even less than the number of isoforms in Ensembl or Gencode annotation. The extreme sparsity of data and the lack of reads capturing an entire transcript prohibit the estimation of isoform-specific gene expression in a single cell. To address this issue, we are currently exploring scRNA-seq data generated using long-read sequencing technologies such as Nanopore and PacBio, to estimate isoform expression in each cell and to detect differential usage of isoforms.

In summary, we have developed a robust approach to detect DAS events by accounting for both technical noise and biological variations such as transcriptional bursting across single cells. Through simulation studies and real data analyses, we showed that SCATS outperformed existing methods in detecting DAS events for both UMI and non-UMI scRNA-seq data. With the increasing adoption of scRNA-seq, we believe SCATS will be well-suited for various splicing studies and offer additional insights beyond total gene expression analysis.

## Materials and methods

### Splicing informative read counts based on exon grouping

To get informative reads for DAS analysis, we pre-process sorted isoform annotation file in GTF format and sorted aligned scRNA-seq data in SAM format. For each gene, alternatively spliced exons are grouped if they originate from the same isoform(s) or complementary isoform(s) (i.e. exons share the same inclusion levels or complementary inclusion levels). Suppose the gene under consideration has *M* exon groups, then all reads mapped to this gene can be grouped into 2*M*+1 categories: 1) uninformative reads: reads that map to exons shared by all isoforms of the gene; 2) included (+) informative reads of exon group *j*: reads mapped to alternatively spliced (AS) exons that are included in exon group *j* (1≤*j*≤*M*); and 3) excluded (-) informative reads of exon group *j*: reads mapped to AS exons that are excluded from exon group *j* (1≤*j*≤*M*).

Let ***Y***_***i***_ = (*Y*_*i*0_,*Y*_+*i*1_,…,*Y*_+*iM*_,*Y*_*−i*1_,…,*Y*_*−iM*_) be the observed read counts in cell *i* for the gene, where *Y*_*i*0_ denotes uninformative read count, *Y*_+*ij*_ denotes included informative read count of exon group *j*, and *Y*_−*ij*_ denotes excluded informative read count of exon group *j*. **Fig 1A** shows an example of how exons are grouped and informative reads are counted. For this hypothetical gene, there are three isoforms constructed by five exons, where three of them are alternatively spliced and grouped into two groups (blue and green). Among the seven mapped reads, reads 1 and 6 are uninformative reads (*Y*_+*i*0_ = 2), read 2 is an informative read included in exon group 1 (*Y*_+*i*1_ = 1), reads 3 and 4 are informative reads excluded from group 1 (*Y*_+*i*0_ = 2), read 5 is an informative read included in exon group 2 (*Y*_+*i*2_ = 1), and read 7 is an informative read excluded from group 2 (*Y*_−*i*2_ = 1). **Fig 1B** shows a workflow that describes how exon grouping and informative read extraction are implemented in SCATS.

### Modeling splicing informative UMI and non-UMI counts

SCATS is designed to detect DAS events at exon group level. For the ease of notation, we drop the exon group index. For a given exon group of interest, let *ψ* be the inclusion level of an included (+) exon group and *θ*_*g*_ be the mean expression of gene *g*. To account for cell-specific technical noise, we model splicing informative read counts *Y*_+*i*_ and *Y*_−*i*_ using a hierarchical model (**S1 Fig**). We assume cell-specific true expression level of the included/excluded exon group *μ*_+*i*_/*μ*_−*i*_ follows a log-normal distribution:

$$\log\left(\mu_{+i}\right)∼Normal(\log\left(\theta_{g}∙\psi ∙h_{+}\right),\sigma_{+}^{2})$$

$$\log\left(\mu_{-i}\right)∼Normal(\log\left(\theta_{g}∙(1-\psi )∙h_{-}\right),\sigma_{-}^{2})$$

where *h*_+_/*h*_−_ represents the probability of included/excluded reads being informative and $\sigma_{+}^{2}/\sigma_{-}^{2}$ is variance of expression level in log scale.

For non-UMI count, amplification bias and low capture efficiency in scRNA-seq are quantified by a linear model. Specifically, for cell *i*, the expected value of the informative read count *λ*_+*i*_/*λ*_−*i*_ is modeled as

$$\log\left(\lambda_{+i}\right)=\alpha_{i}+\beta_{i}\log\left(\mu_{+i}\right)$$

$$\log\left(\lambda_{-i}\right)=\alpha_{i}+\beta_{i}\log\left(\mu_{-i}\right)$$

where *α*_*i*_ and *β*_*i*_ are cell-specific technical parameters designed to model capture efficiency and amplification bias, respectively. These technical parameters can be quantified with TASC[17] when ERCC spike-ins are provided.

For UMI count, cell-specific parameter *β*_*i*_ is equal to 1 as amplification bias can be alleviated by UMI. Therefore, the expected informative UMI count *λ*_+*i*_/*λ*_−*i*_ is proportional to true expression of the included/excluded exon group:

$$\lambda_{+i}=\alpha_{i}\mu_{+i}$$

Moreover, we account for inflated zeros of scRNA-seq which are primarily sourced from two contributing factors: technical dropout and transcriptional bursting. Dropout happens when a particular transcript is lost in scRNA-seq experimental steps while the gene is expressed in the cell. Also, excessive zero counts can be introduced when significant bursting genes are in “off” state due to transcriptional heterogeneity. Previous studies have shown that the modeling of dropout in UMI data is unnecessary[18, 19]. Therefore, to model zero inflation, for a given gene, we let *Z*_*i*_ be the indicator that the gene in cell *i* is captured in the library (dropout does not happen) and it is in the “on” state. The probability of *Z*_*i*_ = 1 is *π*_*i*_. Therefore, given *λ*_+*i*_ and *λ*_−*i*_, the distribution of the observed informative UMI count *Y*_+*i*_/*Y*_−*i*_ is

$$Y_{+i}|Z_{i}=\left\{ \begin{array}{l} \mathrm{Poisson}(\lambda_{+i}),\;if\;Z_{i}=1\\ 0,\;if\;Z_{i}=0 \end{array}\right.$$

and

$$Y_{-i}|Z_{i}=\left\{ \begin{array}{l} \mathrm{Poisson}(\lambda_{-i}),\;\mathrm{if}\;Z_{i}=1\\ 0,\;\mathrm{if}\;Z_{i}=0 \end{array}\right.$$

where Poisson distribution has been reported to approximate the UMI sampling process well after zero inflation being removed[18, 19].

### Probability of included/excluded reads being informative

Another key part of SCATS is to estimate *h*_+_/*h*_−_, the probability of included/excluded reads being splicing informative. From the isoform structure of gene *g*, we can determine the origin (exon group) of a read (being informative read) only when it is mapped to exon-exon junction or an alternatively spliced exon. Thus, a natural way to estimate the probability that a read being mapped to this region is to calculate the ratio between weighted length of the informative region and the whole region. Due to low sequencing depth of scRNA-seq data, we assume the exon group shares the same *h*_+_/*h*_−_ across cells instead of estimating cell-specific parameters:

$${\hat{h}}_{+}=\frac{\sum_{d\in I_{+}}\sum_{s\in S_{d}^{*}}{\hat{\theta }}_{d}w_{s}}{\sum_{d\in I}\sum_{s\in S_{d}}{\hat{\theta }}_{d}w_{s}}$$

$${\hat{h}}_{-}=\frac{\sum_{d\in I_{-}}\sum_{s\in S_{d}^{*}}{\hat{\theta }}_{d}w_{s}}{\sum_{d\in I}\sum_{s\in S_{d}}{\hat{\theta }}_{d}w_{s}}$$

where *I*_+_ and *I*_−_ denote the set of isoforms in the included and excluded group, respectively, *I* denotes the set of all isoforms in gene *g*, $S_{d}^{*}$ denotes the set of informative base pair positions of isoform *d*, and *S*_*d*_ denotes the set of all base pair positions of isoform *d*. To calculate weighted lengths of the region, we utilize observed read coverage *w*_*s*_ at base pair position *s* and relative abundance *θ*_*d*_ of isoform *d* which is estimated based on informative reads. Therefore, the numerator represents the weighted length of informative region for included exon group (+) and excluded exon group (-), respectively, and the denominator represents the weighted length of whole region in gene *g*.

### Detection of differential alternative splicing exon group

Exon-inclusion level, which measures the relative usage of an exon, is a commonly used measure to quantify the alternative splicing process. To detect DAS exons, we can examine if the inclusion level of exon group (*ψ*) is significantly different between two groups of cells. To make accurate inference on the inclusion level difference, technical parameters (*α*_*i*_, *β*_*i*_) for each cell *i* and population mean expression *θ*_*g*_ of gene *g* are first calculated during data pre-processing, using software TASC[17]. This allows us to eliminate bias due to technical variations in scRNA-seq data. Given an exon-group of interest, the informative read counts *Y*_+*i*_/*Y*_−*i*_ are fitted to the hierarchical model described above, with likelihood function calculated as

$$L(\psi |Y_{+1},\ldots ,Y_{+n},Y_{-1},\ldots ,Y_{-n})=\prod_{i=1}^{n}\left[P(Y_{+i}|\psi )P(Y_{-i}|1-\psi )\right]$$

$$=\prod_{i=1}^{n}[\int_{\mu_{+i}}P(Y_{+i},\mu_{+i}|\psi )d\mu_{+i}\int_{\mu_{-i}}P(Y_{-i},\mu_{-i}|\psi )d\mu_{-i}]$$

The joint distribution of cell-specific observed informative read counts and their true expression levels is

$$\begin{array}{l} P(Y_{+i},\mu_{+i}|\psi )=\sum_{Z_{i}=0,1}P(Y_{+i},\mu_{+i}|\psi ,Z_{i})\\ =P(Y_{+i},\mu_{+i}|\psi ,Z_{i}=1)P(Z_{i}=1)+P(Y_{+i},\mu_{+i}|\psi ,Z_{i}=0)P(Z_{i}=0)\\ =\left\{ \begin{array}{l} (1-\pi_{i})f_{LN}(\mu_{+i}|\log\left(\theta_{g}\cdot \psi \cdot h_{+}\right),\sigma_{+}^{2})+e^{-e^{\alpha_{i}+\beta_{i}log\mu_{+i}}}\pi_{i}f_{LN}(\mu_{+i}|\log\left(\theta_{g}\cdot \psi \cdot h_{+}\right),\sigma_{+}^{2}),\;Y_{+i}=0\\ \;\frac{{\left[e^{\alpha_{i}+\beta_{i}log\mu_{+i}}\right]}^{Y_{+i}}e^{-e^{\alpha_{i}+\beta_{i}log\mu_{+i}}}}{Y_{+i}!}\pi_{i}f_{LN}(\mu_{+i}|\log\left(\theta_{g}\cdot \psi \cdot h_{+}\right),\sigma_{+}^{2}),\;Y_{+i}>0 \end{array}\right. \end{array}$$

and similarly

$$P(Y_{-i},\mu_{-i}|\psi )=\left\{ \begin{array}{l} (1-\pi_{i})f_{LN}(\mu_{-i}|\log\left(\theta_{g}\cdot (1-\psi )\cdot h_{-}\right),\sigma_{-}^{2})+e^{-e^{\alpha_{i}+\beta_{i}log\mu_{-i}}}\pi_{i}f_{LN}(\mu_{-i}|\log\left(\theta_{g}\cdot (1-\psi )\cdot h_{-}\right),\sigma_{-}^{2}),\;Y_{-i}=0\\ \;\frac{{\left[e^{\alpha_{i}+\beta_{i}log\mu_{-i}}\right]}^{Y_{-i}}e^{-e^{\alpha_{i}+\beta_{i}log\mu_{-i}}}}{Y_{-i}!}\pi_{i}f_{LN}(\mu_{-i}|\log\left(\theta_{g}\cdot (1-\psi )\cdot h_{-}\right),\sigma_{-}^{2}),\;Y_{-i}>0 \end{array}\right.$$

where the probability distribution function of log-normal distribution $f_{LN}(x|\theta ,\sigma^{2})=\frac{1}{x\sigma \sqrt{2\pi }}e^{-\frac{{\left(\log x-\theta \right)}^{2}}{2\sigma^{2}}}.$

To detect exons with DAS, we test whether there is significant difference in inclusion level *ψ* between two group of cells, denoted by A and B, i.e., *H*_0_:*ψ*_*A*_ = *ψ*_*B*_ vs. *H*_1_:*ψ*_*A*_ ≠ *ψ*_*B*_. A likelihood ratio test with test statistic $T=2|\log\;L_{0}(Y|\hat{\psi })-\log\;L_{1}(Y|\hat{\psi })|$ is employed to test this hypothesis, where $L_{0}(Y|\hat{\psi }),\;L_{1}(Y|\hat{\psi })$ are maximized under the null hypothesis *H*_0_ and alternative hypothesis *H*_1_, respectively. Asymptotically, *T* follows a chi-square distribution with one degree of freedom.

### Datasets and evaluations

We first conducted a simulation study to benchmark the performance of SCATS and compared it with other existing DAS methods, including Census[10] and DEXSeq[13]. To simulate realistic datasets, the average gene expressions (*θ*_*g*_) for each cell group were assigned based on measurements of CA1 pyramidal cells in the scRNA-seq data generated by Zeisel *et al*.[16]. The exon start and end positions were based on Ensembl annotation and downloaded in GTF format from UCSC Genome Browser (https://genome.ucsc.edu/). The cell-specific technical parameters (*α*,*β*,*κ*,*τ*) were estimated based on measurements from CA1 pyramidal cells in scRNA-seq data from Zeisel *et al*.[16]. Given these abundances and technical parameters, we then parameterized the generative model described previously, which allows us to simulate exon-level informative read count (*Y*_+*ij*_,*Y*_−*ij*_) mimicking the scRNA-seq data generation process. Following this procedure, we simulated 1,000 exon groups with 6,000 total reads per cell for 600 cells (300 in condition A, and 300 in condition B) for five scenarios (Δ = |*ψ*_*A*_−*ψ*_*B*_| = 0, 0.1, 0.2, 0.3, 0.4) to evaluate type I error rate and power for SCATS, Census and DEXSeq.

In addition, we designed a non-parametric scRMA-seq simulator without assuming the same model underlying SCATS. First, for each gene, gene-level read count *Y* was assigned based on measurements of CA1 pyramidal cells in the scRNA-seq data generated by Zeisel *et al*. Next, we generated total exon-level informative read counts (*Y*_+_,*Y*_−_) for each condition (A vs B), where *Y*_+_ = *Y*×*ψ*, *Y*_−_ = *Y*×(1−*ψ*). We considered five scenarios (Δ = |*ψ*_*A*_−*ψ*_*B*_| = 0, 0.1, 0.2, 0.3, 0.4). Given total informative read counts (*Y*_+_,*Y*_−_), we then simulated cell-specific informative read counts (*Y*_+i_,*Y*_−i_) non-parametrically. Specifically, for each single cell *i*, the informative read counts (*Y*_+i_,*Y*_−i_) were generated from multinomial distributions: *Y*_+*i*_~multinomial(*Y*_+_,***p***) and *Y*_−*i*_~multinomial(*Y*_−_,***p***), where ***p***~*Dirichlet*(***α***) and ***α*** = (*α*_1_,…,*α*_300_) = (10,…,10).

Next, we evaluated different DAS detection methods using mouse cortical dataset generated by Tasic *et al*.[3], which includes 1,679 cells from 49 sub-cell types in three major cell classes (23 GABAergic cell types, 19 glutamatergic cell types, and seven non-neuronal cell types). Among these cells, the average sequencing depth is 3 million. We directly downloaded the aligned data in BAM format from NCBI Gene Expression Omnibus (GSE71585). Given read counts of 6,275 endogenous RefSeq genes with at least two annotated isoforms and 92 ERCC spike-ins across cells, we applied TASC to quantify technical variations, yielding accurate estimation of average gene concentrations in each sub-cell type. Similarly, informative reads in each exon group were counted using SCATS based on the 6,275 genes with 12,073 exon groups. On average, 4,311 exon groups were covered by at least one splicing informative read in each cell. We treat an exon group as qualified for analysis when at least three cells have at least one informative read in each condition. Pairwise DAS comparison was conducted between these 49 sub-cell types with 985 qualified exon groups on average for each comparison.

Another adult mouse brain data used for evaluation was acquired from Zeisel *et al*.[16], which includes sequencing data of 3,005 cells from various regions of mouse brain. These cells were classified into nine major cell classes and 47 sub-cell types with sub-cell types from the same major cell class considered to be relatively homogenous. In this study, raw data in FASTQ format were downloaded from NCBI Gene Expression Omnibus (GSE60361) and aligned to mm10 reference genome with Ensembl gene annotation using STAR[20]. For each cell, we processed the BAM file and counted splicing informative reads using SCATS for 1,826 marker genes (with at least two annotated isoforms by Ensembl) with 3,542 exon groups of the nine major cell classes. For each cell, there are 465 exon groups on average covered by at least one splicing informative UMI read. Given these informative counts, DAS analysis between each pair of the 47 sub-cell types was conducted. To compare the ability of DAS detection, we also performed pairwise DAS analysis across sub-cell types using DEXSeq and Census. For each comparison, we required at least three cells having at least one informative UMI in each condition, leading to 568 qualified exon groups on average for each DAS comparison.

### Software availability

An open-source implementation of the SCATS algorithm can be downloaded from https://github.com/huyustats/SCATS.

### Life sciences reporting summary

Further information on experimental design is available in the Life Sciences Reporting Summary.

## Supporting information

S1 Fig Schematic of the SCATS model. An alternatively spliced exon is included in the “+” exon group (blue) and excluded from the “-” group (white). *μ*_+*i*_ and *μ_−i_* represent true expression levels of the “+” and “-” exon groups, respectively, in cell *i*. *λ*_+*i*_ and *λ*_−*i*_ are intermediate variables that model amplification bias, capture efficiency, and sequencing bias in cell *i* for the “+” and “-”exon groups. *Z*_*i*_ models transcriptional bursting and dropout event of the gene in cell *i*. *Y*_+*i*_ and *Y*_−*i*_ represent observed informative read counts of the “+” and “-” exon groups in cell *i*. SCATS utilizes a hierarchical model to detect differential alternative splicing (DAS) events between cell groups by accounting for technical noise from scRNA-seq data.
(TIF)

S2 Fig Results from a simulation study based on nonparametric scRNA-seq simulator. **(A)** Quantile-quantile plots of the p-values from SCATS, Census and DEXSeq under the null hypothesis (Δ = 0). X-axis represents uniform theoretical quantiles between 0 and 1 in −log_10_ scale. Y-axis represents observed p-value quantile in –log_10_ scale. Uniformly distributed data should follow the red dashed line. P-values of SCATS are more uniformly distributed while those from Census are right-skewed and those from DEXSeq are left-skewed. **(B)** Type I error comparison of SCATS, Census and DEXSeq with different significance levels (α = 0.05, 0.01, 0.005). Consistent with **(B)**, SCATS has better type I error control than Census and DEXSeq. **(C)** Barplots show the estimated power under different effect sizes (Δ = 0.1, 0.2, 0.3, 0.4). Significance was evaluated at 0.05, 0.01, and 0.005 levels, respectively. SCATS outperformed Census and DEXSeq across all effect sizes, especially when Δ = 0.4. DEXSeq is conservative in detecting DAS events.
(TIF)

S3 Fig Impact of isoform under-annotation on SCATS. Simulated scRNA-seq read counts were based on 100% Ensembl annotated isoforms, but analyzed with SCATS using 100%, 75% and 50% of the annotated isoforms. Barplots show the estimated power under different effect sizes (Δ = 0.1, 0.2, 0.3, 0.4). Significance was evaluated at 0.05, 0.01, and 0.005 levels, respectively. The performance of SCATS is robust to under-annotation of isoform.
(TIF)

S4 Fig Impact of exon group length on SCATS. Exon groups were divided into three groups: length<130bp, 130bp≤length<388bp, and length≥388bp. Barplots show the estimated power of SCATS under different effect sizes (Δ = 0.1, 0.2, 0.3, 0.4). Significance was evaluated at the 0.05, 0.01, and 0.005 levels.
(TIF)

S5 Fig Comparison between SCATS and pseudo-bulk approach based on data simulated from the generative model. Barplots show the estimated power of SCATS and pseudo-bulk approach under different effect sizes (Δ = 0.1, 0.2, 0.3, 0.4). Significance was evaluated at the 0.05 level for SCATS. For the pseudo-bulk data, since there is only one sample per condition, we cannot perform a statistical test. To evaluate its performance, we declared an event to be significant if the fold change (FC) of exon-inclusion level is greater than 1.2 (FC>1.2)
(TIF)

S6 Fig DAS analysis of the AMPA receptor genes, *Gria1* and *Gria2* using Tasic *et al*. dataset. **(A,B)** Flip-flop exons of genes *Gria1* **(A)** and *Gria2* **(B)**. **(C,D)** Heatmaps show the p-values of pairwise DAS tests for the flop exon for *Gria1* **(C)** and *Gria2* **(D)** across 16 cell types (8 GABAergic, 7 Glutamatergic, 1 non-neuronal) using SCATS. These 16 cell types were selected by Tasic *et al*. in which they found highly cell type-specific splicing patterns across these cell types. As expected, SCATS results also showed highly cell type-specific splicing pattern of the *Gria* genes.
(TIF)

S7 Fig Quantile-quantile plots of p-values from SCATS, DEXSeq and Census using the Tasic *et al*. dataset. Pairwise DAS comparison across 49 sub-cell types in three major cell classes was performed using SCATS. DAS comparisons were classified into two groups: comparison for cell types within major cell classes **(A, C, E)**, and comparison for cell types across major cell classes **(B, D, F)**. X-axis represents uniform theoretical quantiles between 0 and 1 in −log_10_ scale. Y-axis represents observed p-value quantile in −log_10_ scale. Uniformly distributed data should follow the red dashed line. Q-Q plots of p-values from within major cell class comparisons (GABAergic, Glutamatergic, Non-neuronal) are similar to the plot under the null hypothesis in our simulation study. This matched our expectation because most exons should not be differentially spliced between sub-cell types within the same major cell class. In contrast, the distribution of p-values is highly right-skewed starting from X = 10^−1.5^ for cross-major cell class comparisons (GABAergic vs. Glutamatergic, GABAergic vs. Non-neuronal, Glutamatergic vs. Non-neuronal), indicating that more DAS events were detected.
(TIF)

S8 Fig DAS analysis results of the Tasic *et al*. data based on 296 genes with 966 exon groups. The 296 genes were detected as the most differentially spliced genes by Tasic *et al*. based on MISO analysis. **(A-F)** Pairwise DAS comparison across 49 sub-cell types from three major cell classes: GABAergic, Glutamatergic, and Non-neuronal from mouse cortex using SCATS **(A,B)**, Census **(C,D)** and DEXSeq **(E,F)**. Colors indicate different sub-cell types. **(A,C,E)** Heatmaps showing the proportion of detected DAS exon groups for each pairwise comparison between sub-cell types. **(B,D,F)** Dendrograms depicting cell classification results of the 49 sub-cell types. Each sub-cell type was marked by the corresponding major cell class: GABAergic (red), Glutamatergic (blue) and Non-neuronal (grey). The distance metric between two sub-cell types is the proportion of detected DAS exon groups for SCATS **(B)**, and DAS exons for Census **(D)** and DEXSeq **(F)**.
(TIF)

S9 Fig Informative read coverage of flip-flop exons of *Gria1* based on Tasic *et al*. data. **(A)** IGV sashimi plot of the flip-flop exons between cell types Sst Cdk6 and Sst Cbln4. These differentially spliced flip-flop exons were identified by SCATS but missed by Census and DEXSeq. Each row represents one single cell. Six cells were randomly selected from the 68 Sst Cbln4 cell, and another six cells were randomly selected from the 19 Sst Cdk6 cells. **(B)** Scatter plot of the flip exon coverage against the flop exon coverage across all 87 cells. Black dashed line is identical line. The read coverage showed a significant difference in flip-flop exon usage between cell types Sst Cdk6 and Sst Cbln4.
(TIF)

S10 Fig DAS analysis results of 6,275 genes meeting analysis criteria in the Tasic *et al*. data. Pairwise DAS comparison across GABAergic cell types from mouse cortex using SCATS, Census and DEXSeq. Colors indicate different GABAergic cell types. Purple, yellow and red indicate three sub cell classes. Heatmaps showing the proportions of detected DAS exon groups or exons for each pairwise comparison between cell types based on 6,275 genes. Dendrograms depicting cell classification results of the 17 GABAergic cell types. The distance metric between two cell types is the proportion of detected DAS exon groups for SCATS, and DAS exons for Census and DEXSeq. SCATS outperformed Census and DEXSeq in detecting splicing heterogeneity across different cell types, while controlling false positive rate.
(TIF)

S11 Fig DAS analysis results of the Zeisel *et al*. data. This dataset includes both UMI and non-UMI read counts. The UMI read counts were obtained by consolidating read counts for reads that originate from the same molecule based on UMI barcodes. This dataset allows us to compare UMI and non-UMI read counts systematically for DAS analysis. **(A,B)** Comparison of UMI and non-UMI read counts. **(A)** Scatter plot of UMI splicing informative read counts against non-UMI splicing informative read counts in log scale. Each point was summarized based on the same exon group in the same cell. Red dashed line represents identical line. **(B)** Distribution comparison between UMI and non-UMI splicing informative read counts. UMI counts are much smaller than non-UMI counts, suggesting that splicing analysis using UMI counts is more challenging. **(C,D)** Pairwise DAS comparison across nine major cell types from mouse cortex and hippocampus using MISO. Colors indicate nine major cell classes. **(C)** Heatmaps showing the proportion of detected DAS exon groups for each pairwise comparison between sub-cell types. **(D)** Dendrogram depicting cell classification results of the 47 sub-cell types. The distance metric between two sub-cell types is the proportion of detected DAS exons among all analyzed exons by MISO. MISO showed the worst performance in cell type classification as compared to SCATS, Census and DEXSeq in **Fig 3**. **(E)** Gene-level UMI counts and exon-inclusion level estimates of the flip exon from gene *Gria1* across sub-cell types. Splicing quantification offers higher resolution of cellular heterogeneity than total gene expression.
(TIF)

S12 Fig DAS analysis comparison across different significance levels and methods. The analysis was conducted on 296 genes detected as the most differentially spliced genes by Tasic *et al*. based on MISO analysis. Heatmaps showing the proportions of detected DAS events for each pairwise comparison between 49 sub-cell types. The performance of SCATS, Census and DEXSeq was evaluated using different significance levels (α = 0.05, 0.01, 0.005). For within major cell class comparison (GABAergic, Glutamatergic, Non-neuronal), Census yielded much higher DAS detection rate (~0.6) than SCATS (<0.1), indicating possible inflated false positive results for Census as we expect to see smaller splicing differences for cells within the same major cell class.
(TIF)

S13 Fig DAS analysis comparison across different significance levels and methods using Zeisel *et al*. data. Heatmaps showing the proportion of detected DAS events (3,542 exon groups from 1,826 genes) for each pairwise comparison between nine major cell classes. The performance of SCATS, Census, DEXSeq and MISO was evaluated at different significance levels (α = 0.05, 0.01, 0.005 for SCATS, Census and DEXSeq, or Bayes factor: 50, 100, 200 for MISO).
(TIF)
