# Supplementary figures and images for: Detecting differential alternative splicing events in scRNA-seq with or without Unique Molecular Identifiers
Source: PLoS Comput Biol. 2020 Jun 5;16(6):e1007925. doi: 10.1371/journal.pcbi.1007925 (PMC7299405; doi:10.1371/journal.pcbi.1007925)

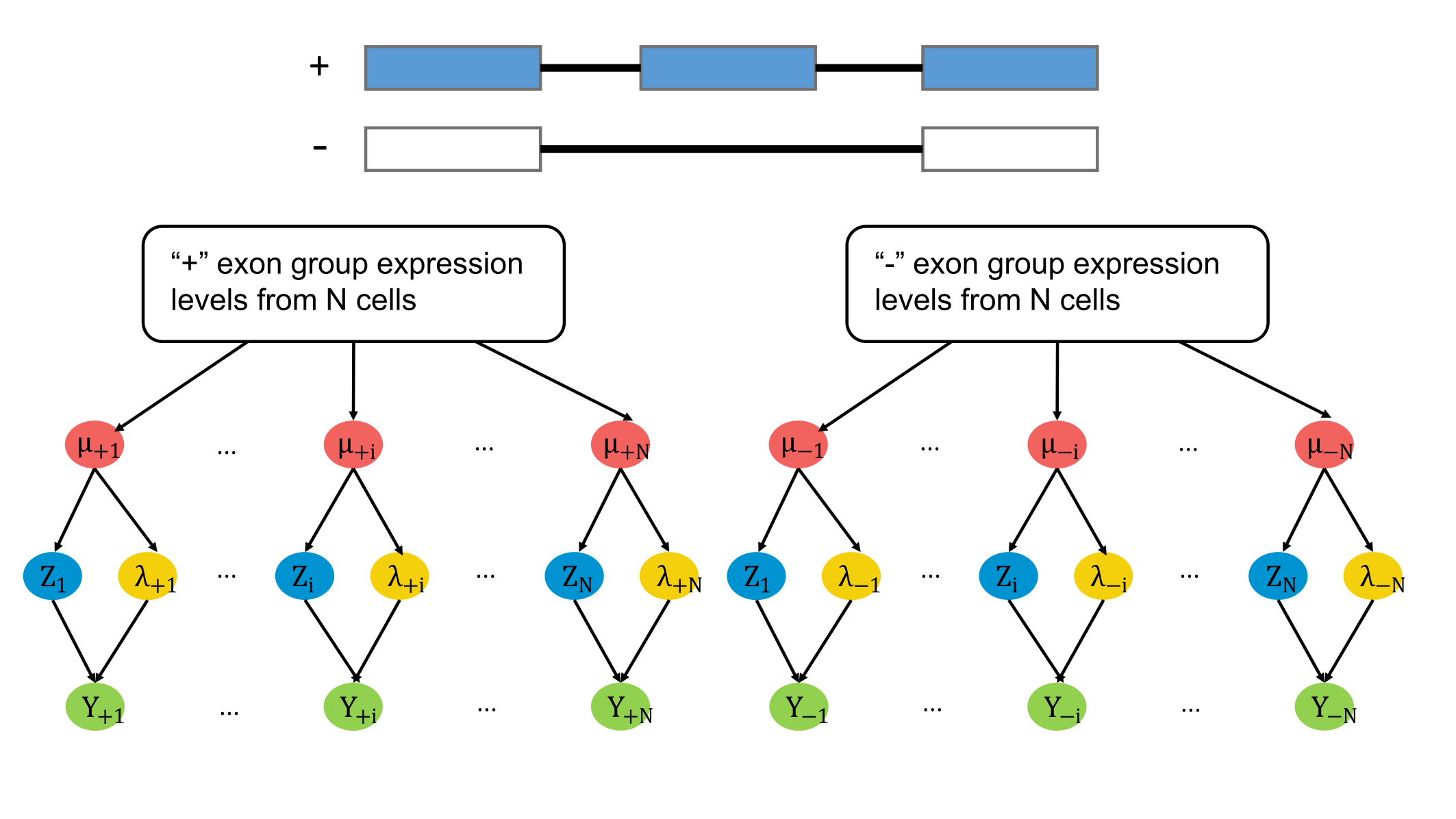

Supplement: S1 Fig — An alternatively spliced exon is included in the “+” exon group (blue) and excluded from the “-” group (white). μ+i and μ−i represent true expression levels of the “+” and “-” exon groups, respectively, in cell i. λ+i and λ−i are intermediate variables that model amplification bias, capture efficiency, and sequencing bias in cell i for the “+” and “-”exon groups. Zi models transcriptional bursting and dropout event of the gene in cell i. Y+i and Y−i represent observed informative read counts of the “+” and “-” exon groups in cell i. SCATS utilizes a hierarchical model to detect differential alternative splicing (DAS) events between cell groups by accounting for technical noise from scRNA-seq data. (TIF) [file pcbi.1007925.s001.tif]

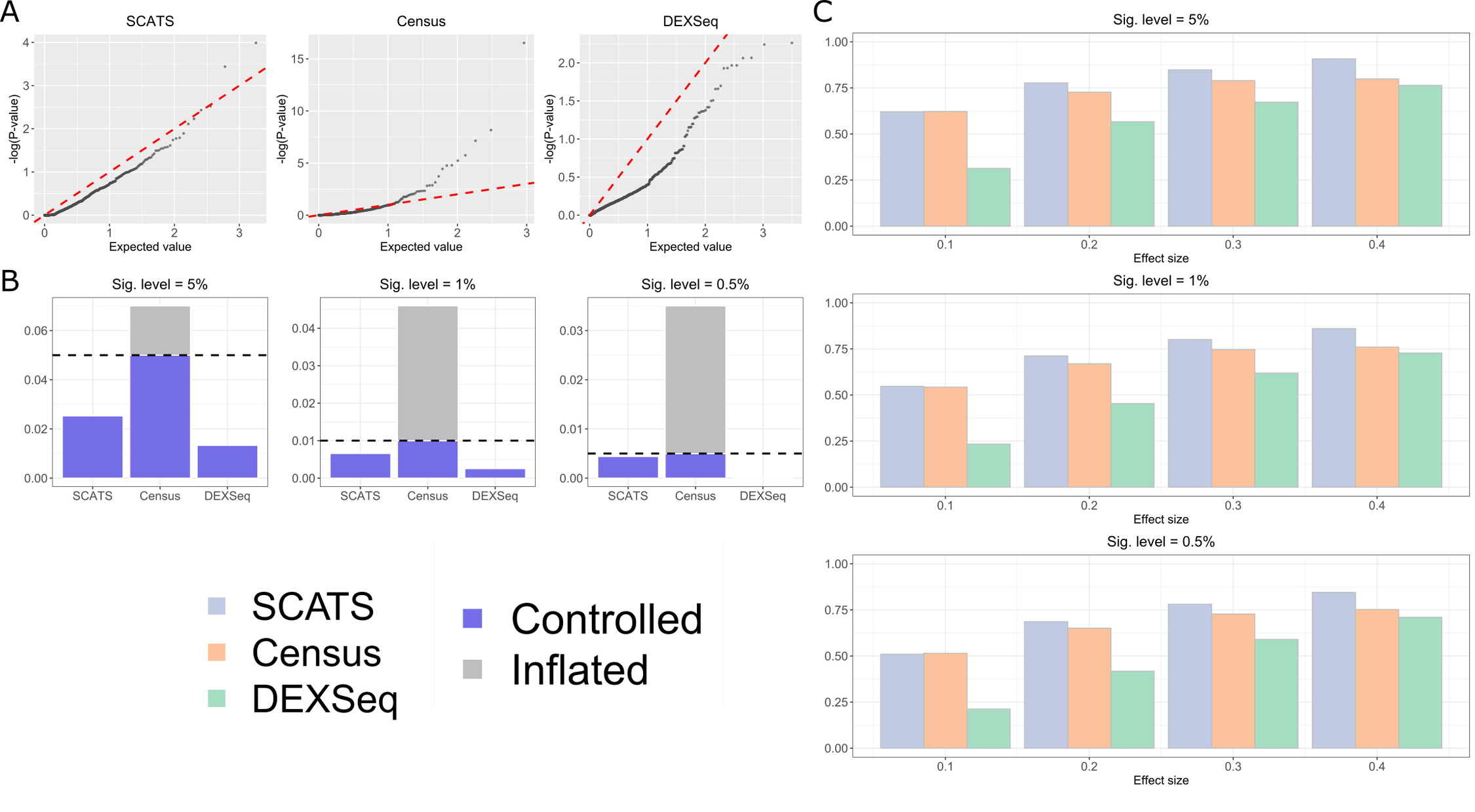

Supplement: S2 Fig — (A) Quantile-quantile plots of the p-values from SCATS, Census and DEXSeq under the null hypothesis (Δ = 0). X-axis represents uniform theoretical quantiles between 0 and 1 in −log10 scale. Y-axis represents observed p-value quantile in –log10 scale. Uniformly distributed data should follow the red dashed line. P-values of SCATS are more uniformly distributed while those from Census are right-skewed and those from DEXSeq are left-skewed. (B) Type I error comparison of SCATS, Census and DEXSeq with different significance levels (α = 0.05, 0.01, 0.005). Consistent with (B), SCATS has better type I error control than Census and DEXSeq. (C) Barplots show the estimated power under different effect sizes (Δ = 0.1, 0.2, 0.3, 0.4). Significance was evaluated at 0.05, 0.01, and 0.005 levels, respectively. SCATS outperformed Census and DEXSeq across all effect sizes, especially when Δ = 0.4. DEXSeq is conservative in detecting DAS events. (TIF) [file pcbi.1007925.s002.tif]

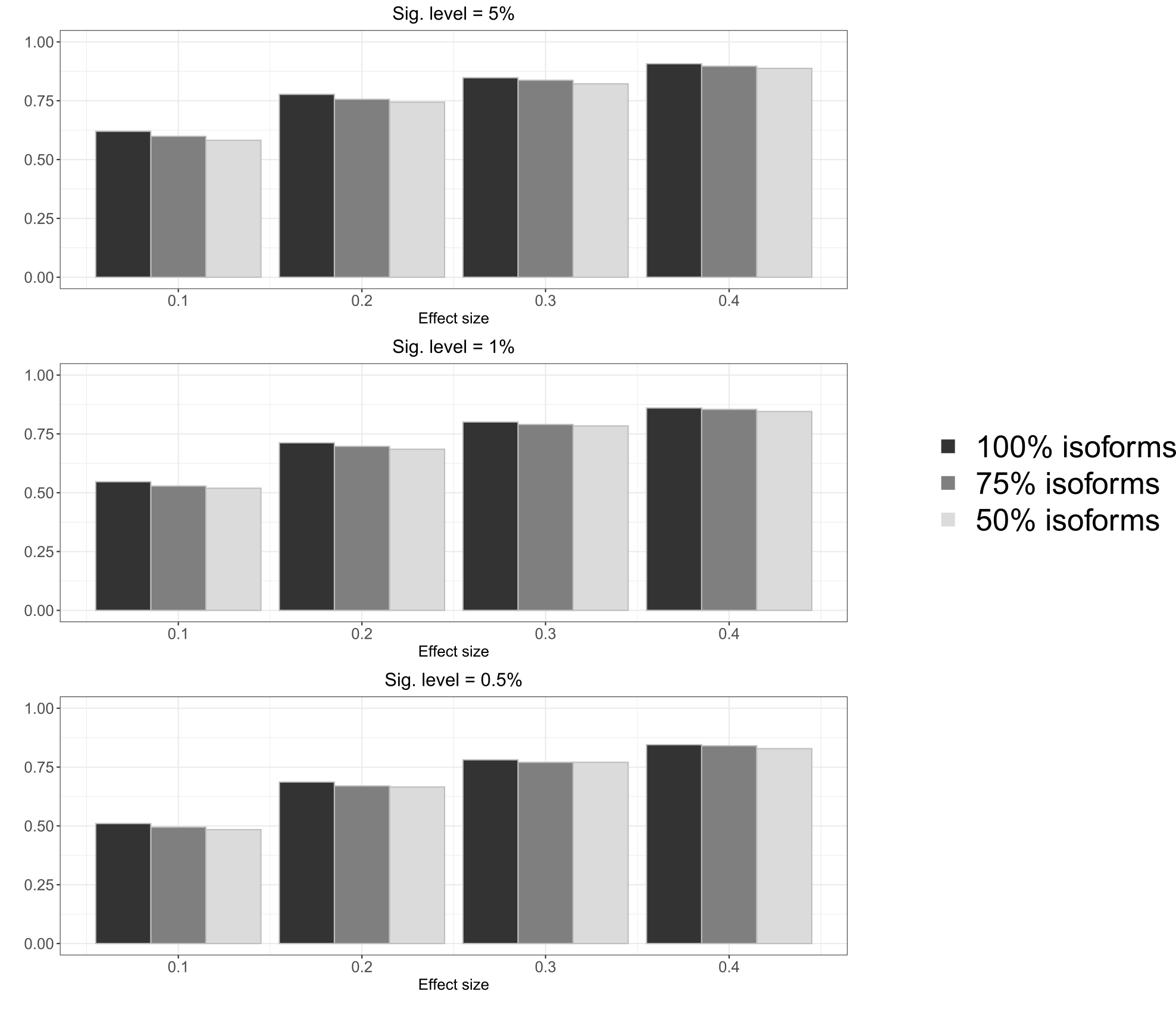

Supplement: S3 Fig — Simulated scRNA-seq read counts were based on 100% Ensembl annotated isoforms, but analyzed with SCATS using 100%, 75% and 50% of the annotated isoforms. Barplots show the estimated power under different effect sizes (Δ = 0.1, 0.2, 0.3, 0.4). Significance was evaluated at 0.05, 0.01, and 0.005 levels, respectively. The performance of SCATS is robust to under-annotation of isoform. (TIF) [file pcbi.1007925.s003.tif]

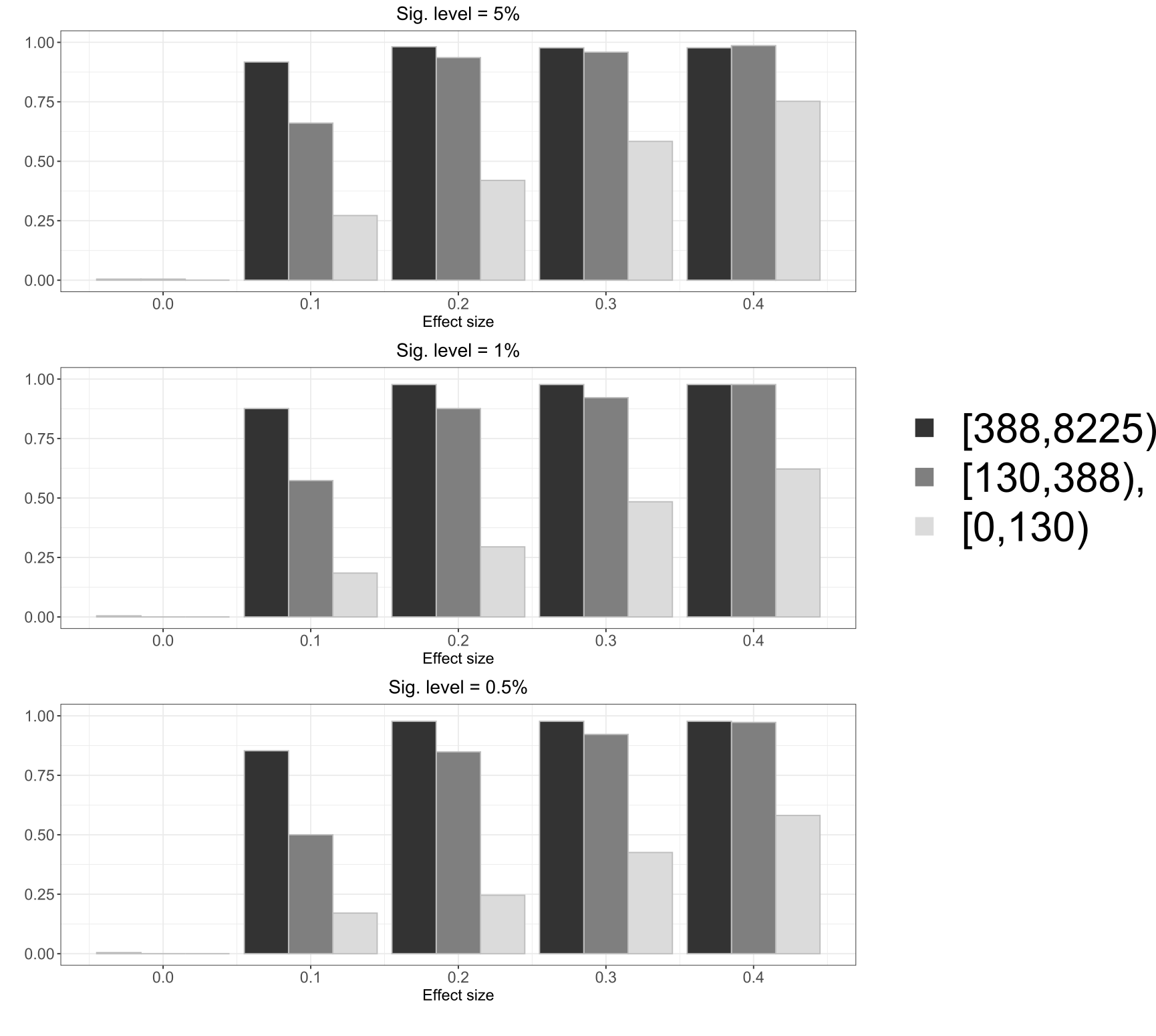

Supplement: S4 Fig — Exon groups were divided into three groups: length<130bp, 130bp≤length<388bp, and length≥388bp. Barplots show the estimated power of SCATS under different effect sizes (Δ = 0.1, 0.2, 0.3, 0.4). Significance was evaluated at the 0.05, 0.01, and 0.005 levels. (TIF) [file pcbi.1007925.s004.tif]

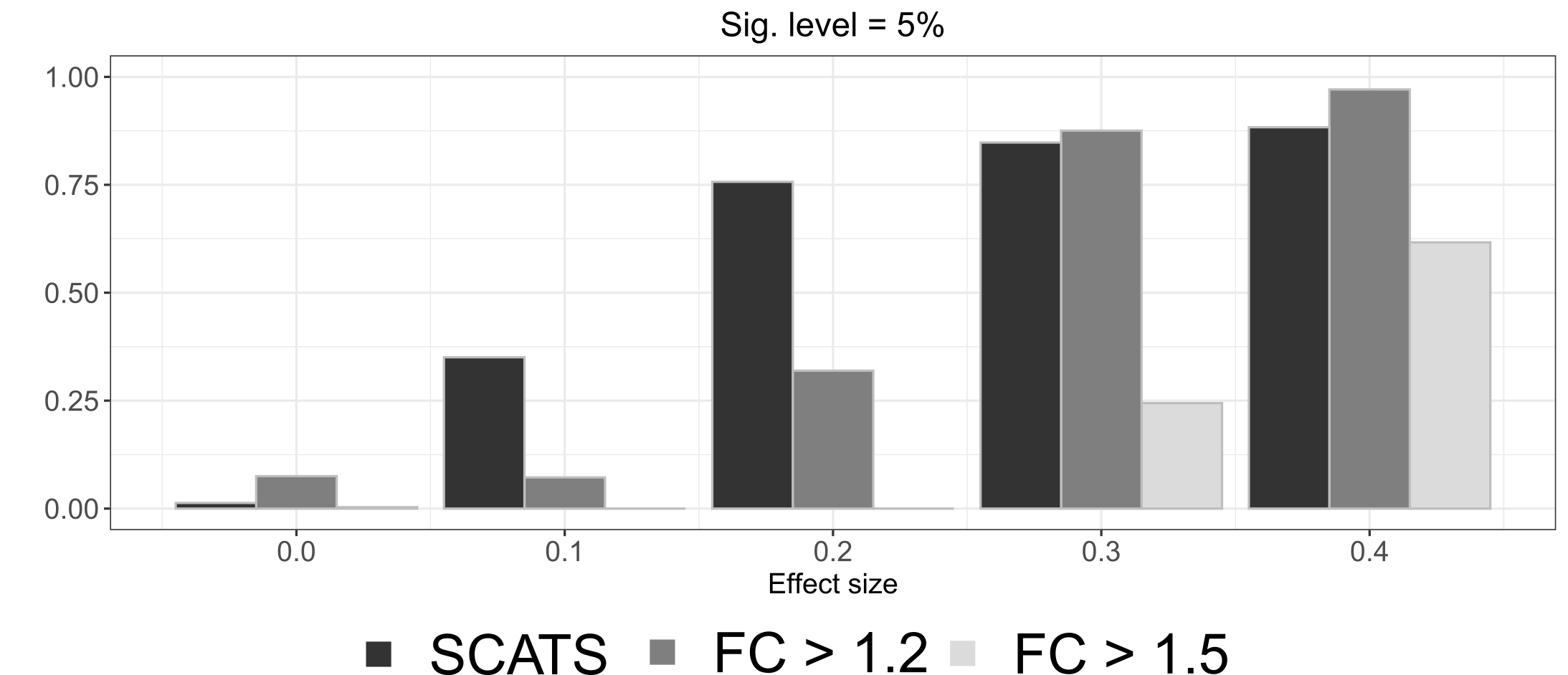

Supplement: S5 Fig — Barplots show the estimated power of SCATS and pseudo-bulk approach under different effect sizes (Δ = 0.1, 0.2, 0.3, 0.4). Significance was evaluated at the 0.05 level for SCATS. For the pseudo-bulk data, since there is only one sample per condition, we cannot perform a statistical test. To evaluate its performance, we declared an event to be significant if the fold change (FC) of exon-inclusion level is greater than 1.2 (FC>1.2) (TIF) [file pcbi.1007925.s005.tif]

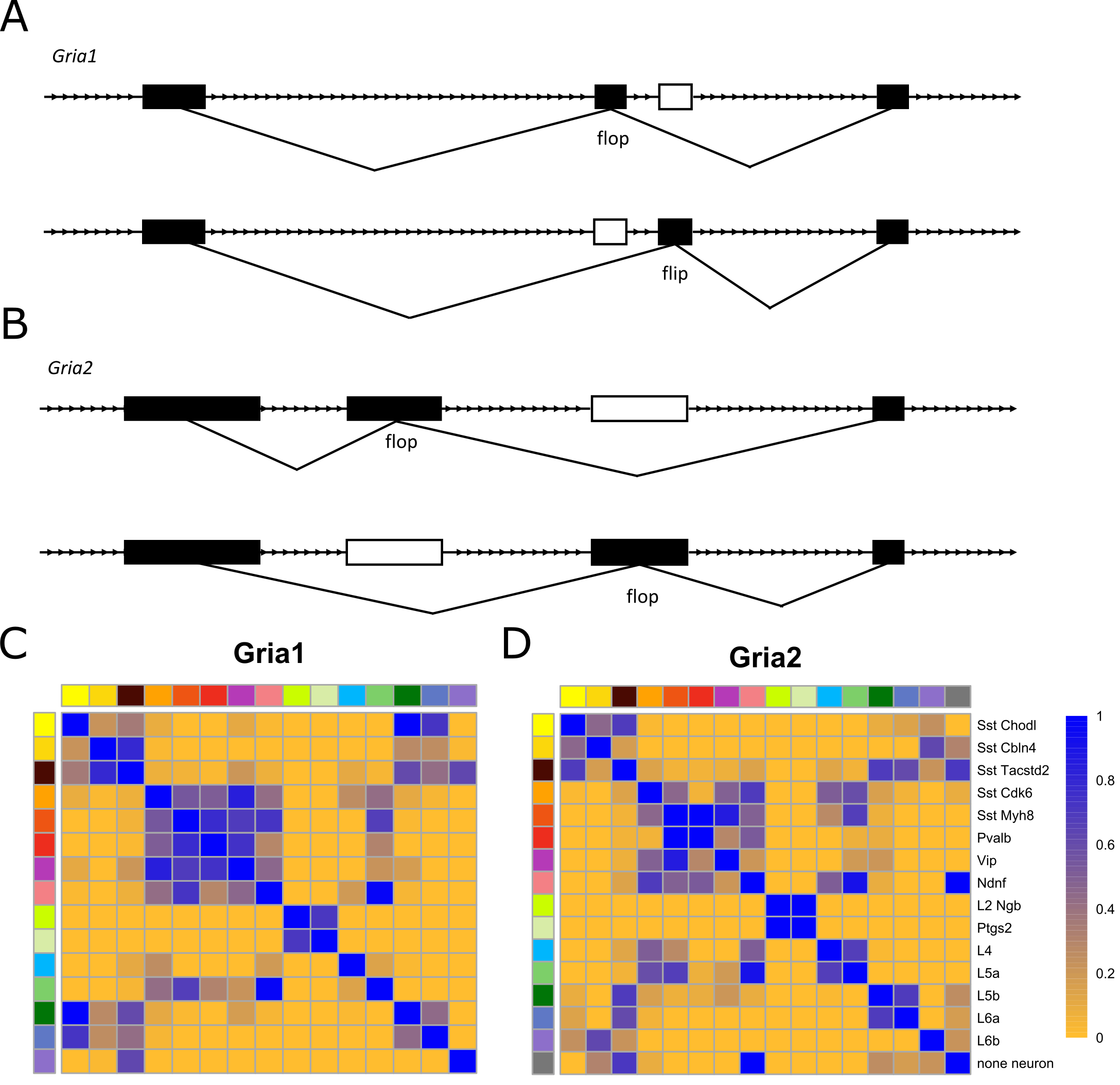

Supplement: S6 Fig — (A,B) Flip-flop exons of genes Gria1 (A) and Gria2 (B). (C,D) Heatmaps show the p-values of pairwise DAS tests for the flop exon for Gria1 (C) and Gria2 (D) across 16 cell types (8 GABAergic, 7 Glutamatergic, 1 non-neuronal) using SCATS. These 16 cell types were selected by Tasic et al. in which they found highly cell type-specific splicing patterns across these cell types. As expected, SCATS results also showed highly cell type-specific splicing pattern of the Gria genes. (TIF) [file pcbi.1007925.s006.tif]

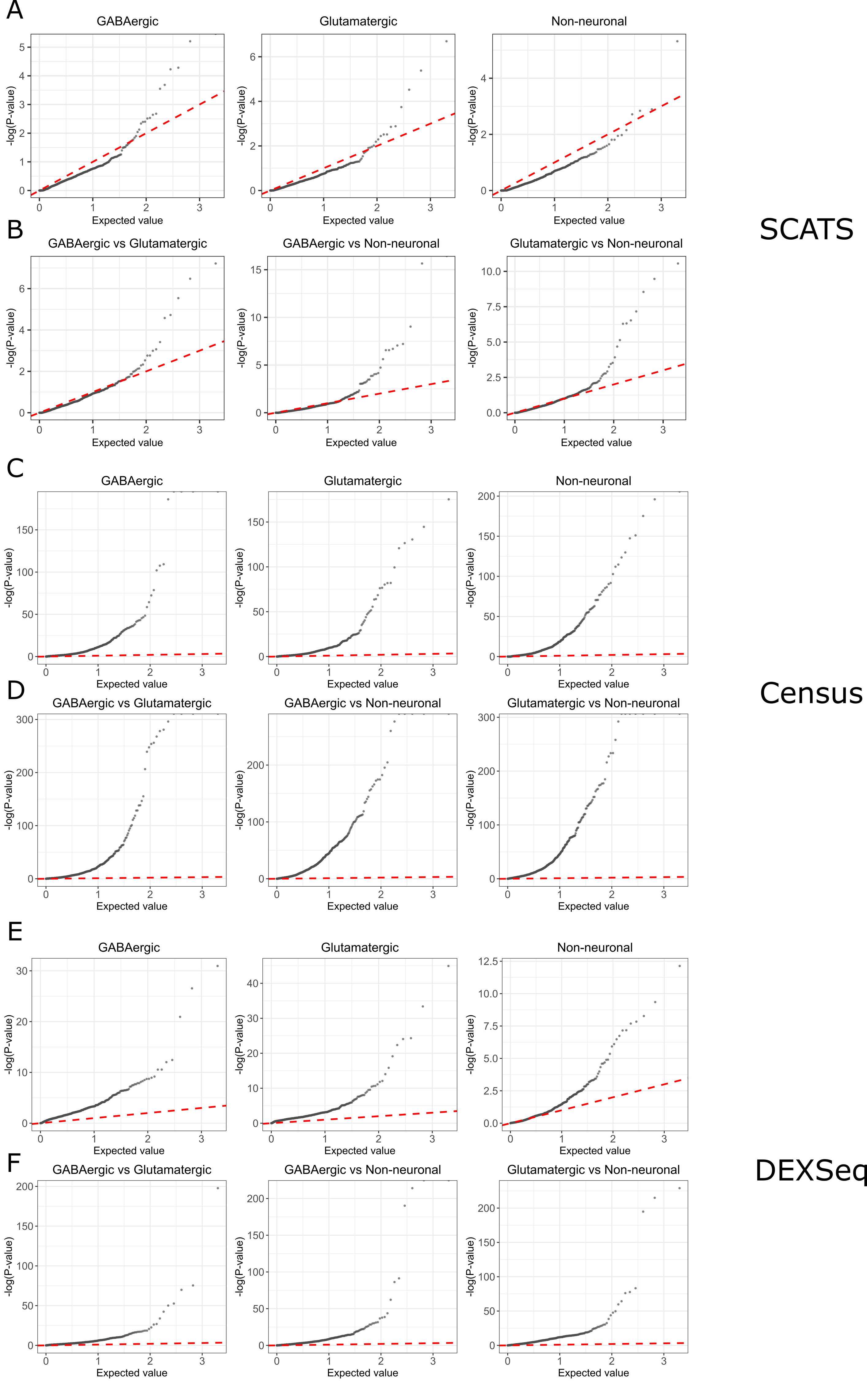

Supplement: S7 Fig — Pairwise DAS comparison across 49 sub-cell types in three major cell classes was performed using SCATS. DAS comparisons were classified into two groups: comparison for cell types within major cell classes (A, C, E), and comparison for cell types across major cell classes (B, D, F). X-axis represents uniform theoretical quantiles between 0 and 1 in −log10 scale. Y-axis represents observed p-value quantile in −log10 scale. Uniformly distributed data should follow the red dashed line. Q-Q plots of p-values from within major cell class comparisons (GABAergic, Glutamatergic, Non-neuronal) are similar to the plot under the null hypothesis in our simulation study. This matched our expectation because most exons should not be differentially spliced between sub-cell types within the same major cell class. In contrast, the distribution of p-values is highly right-skewed starting from X = 10−1.5 for cross-major cell class comparisons (GABAergic vs. Glutamatergic, GABAergic vs. Non-neuronal, Glutamatergic vs. Non-neuronal), indicating that more DAS events were detected. (TIF) [file pcbi.1007925.s007.tif]

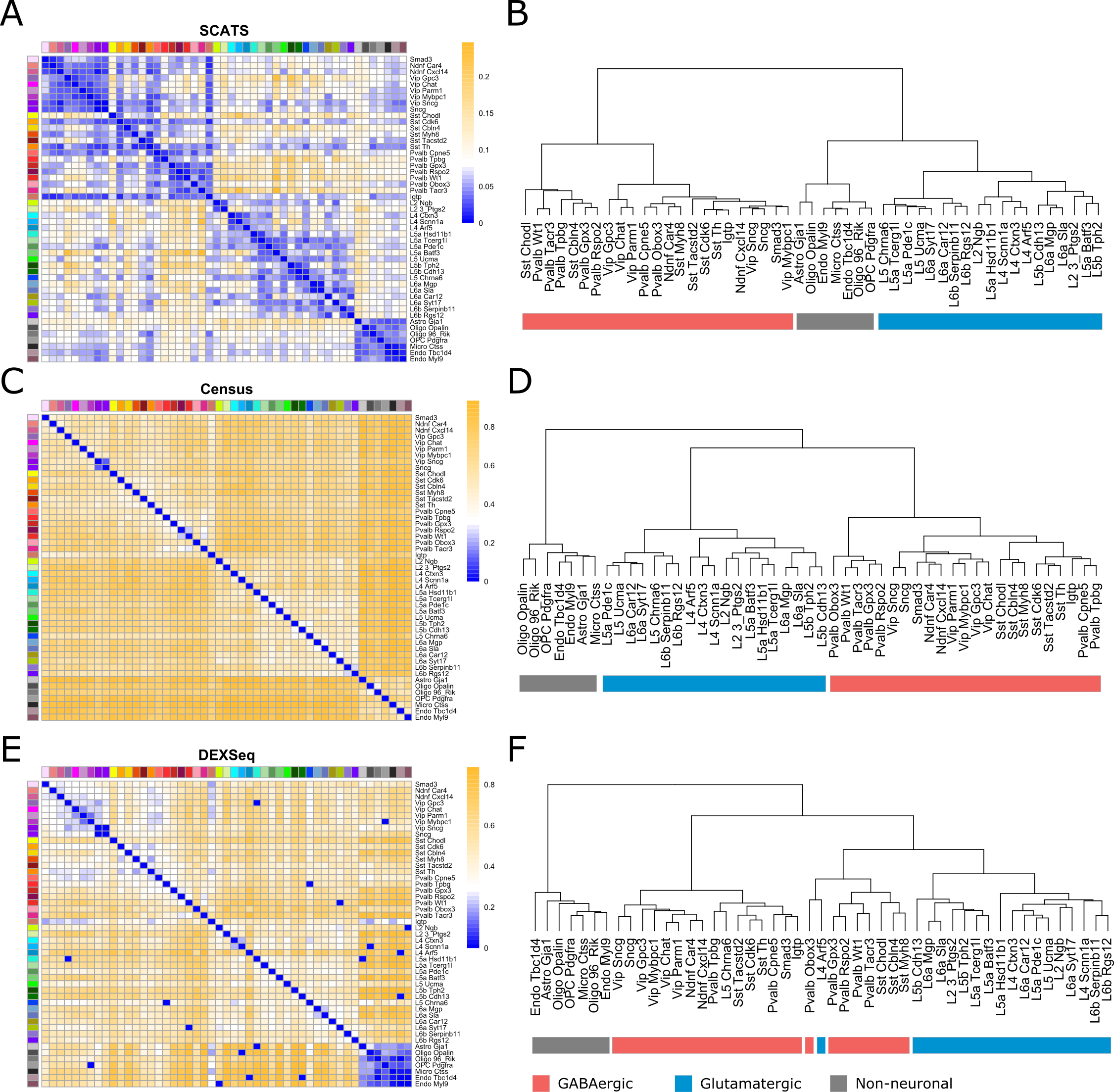

Supplement: S8 Fig — The 296 genes were detected as the most differentially spliced genes by Tasic et al. based on MISO analysis. (A-F) Pairwise DAS comparison across 49 sub-cell types from three major cell classes: GABAergic, Glutamatergic, and Non-neuronal from mouse cortex using SCATS (A,B), Census (C,D) and DEXSeq (E,F). Colors indicate different sub-cell types. (A,C,E) Heatmaps showing the proportion of detected DAS exon groups for each pairwise comparison between sub-cell types. (B,D,F) Dendrograms depicting cell classification results of the 49 sub-cell types. Each sub-cell type was marked by the corresponding major cell class: GABAergic (red), Glutamatergic (blue) and Non-neuronal (grey). The distance metric between two sub-cell types is the proportion of detected DAS exon groups for SCATS (B), and DAS exons for Census (D) and DEXSeq (F). (TIF) [file pcbi.1007925.s008.tif]

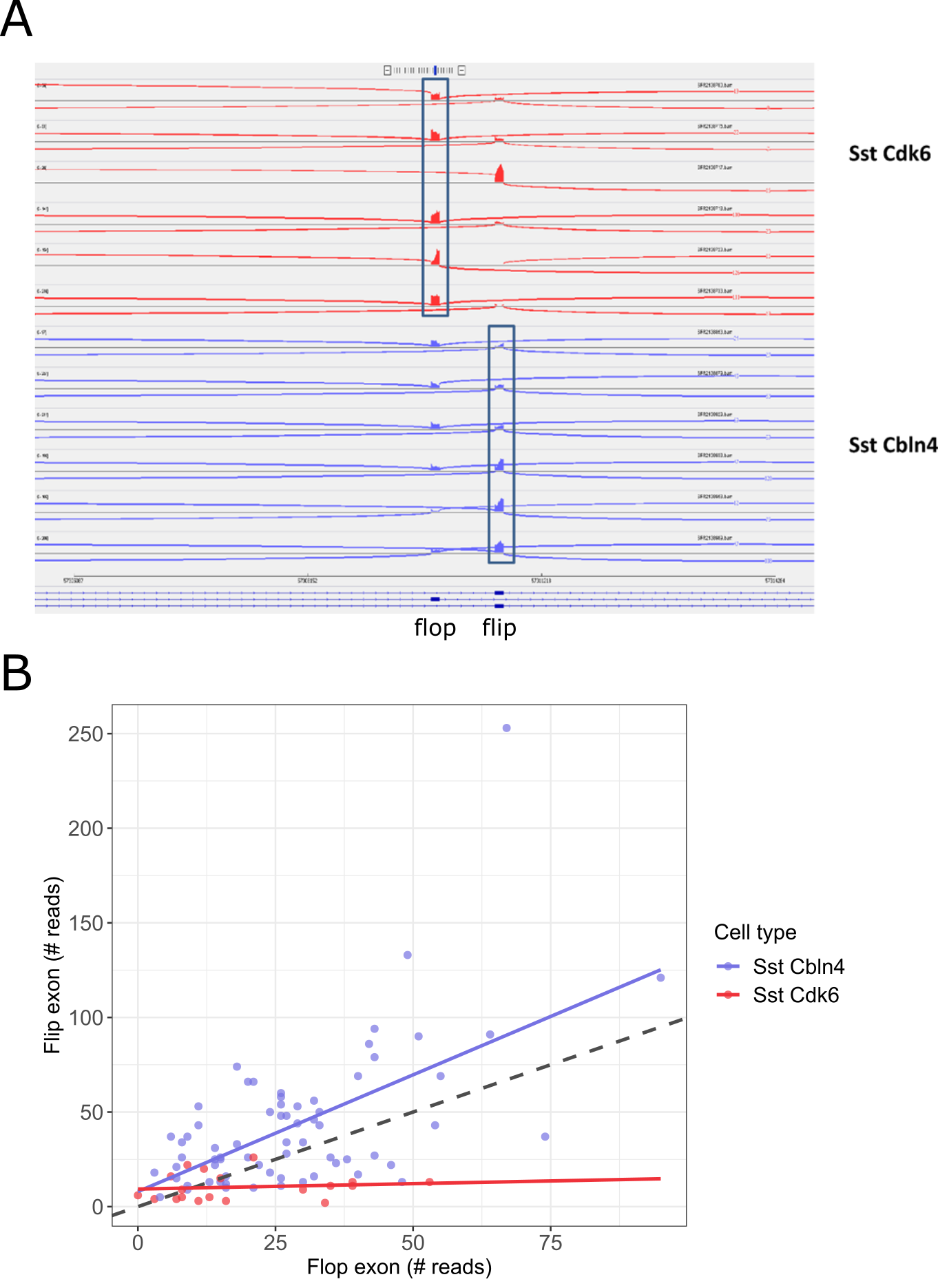

Supplement: S9 Fig — (A) IGV sashimi plot of the flip-flop exons between cell types Sst Cdk6 and Sst Cbln4. These differentially spliced flip-flop exons were identified by SCATS but missed by Census and DEXSeq. Each row represents one single cell. Six cells were randomly selected from the 68 Sst Cbln4 cell, and another six cells were randomly selected from the 19 Sst Cdk6 cells. (B) Scatter plot of the flip exon coverage against the flop exon coverage across all 87 cells. Black dashed line is identical line. The read coverage showed a significant difference in flip-flop exon usage between cell types Sst Cdk6 and Sst Cbln4. (TIF) [file pcbi.1007925.s009.tif]

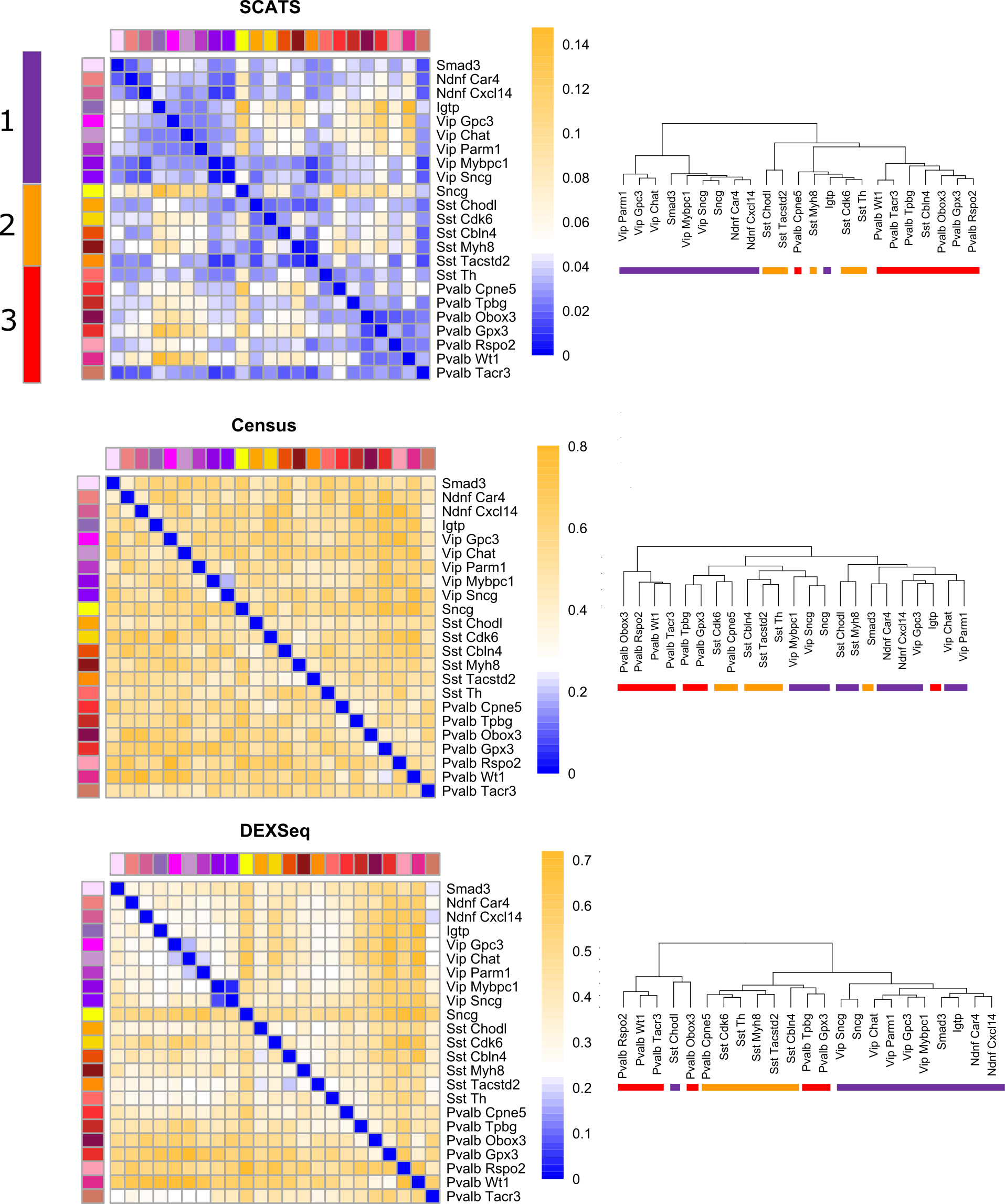

Supplement: S10 Fig — Pairwise DAS comparison across GABAergic cell types from mouse cortex using SCATS, Census and DEXSeq. Colors indicate different GABAergic cell types. Purple, yellow and red indicate three sub cell classes. Heatmaps showing the proportions of detected DAS exon groups or exons for each pairwise comparison between cell types based on 6,275 genes. Dendrograms depicting cell classification results of the 17 GABAergic cell types. The distance metric between two cell types is the proportion of detected DAS exon groups for SCATS, and DAS exons for Census and DEXSeq. SCATS outperformed Census and DEXSeq in detecting splicing heterogeneity across different cell types, while controlling false positive rate. (TIF) [file pcbi.1007925.s010.tif]

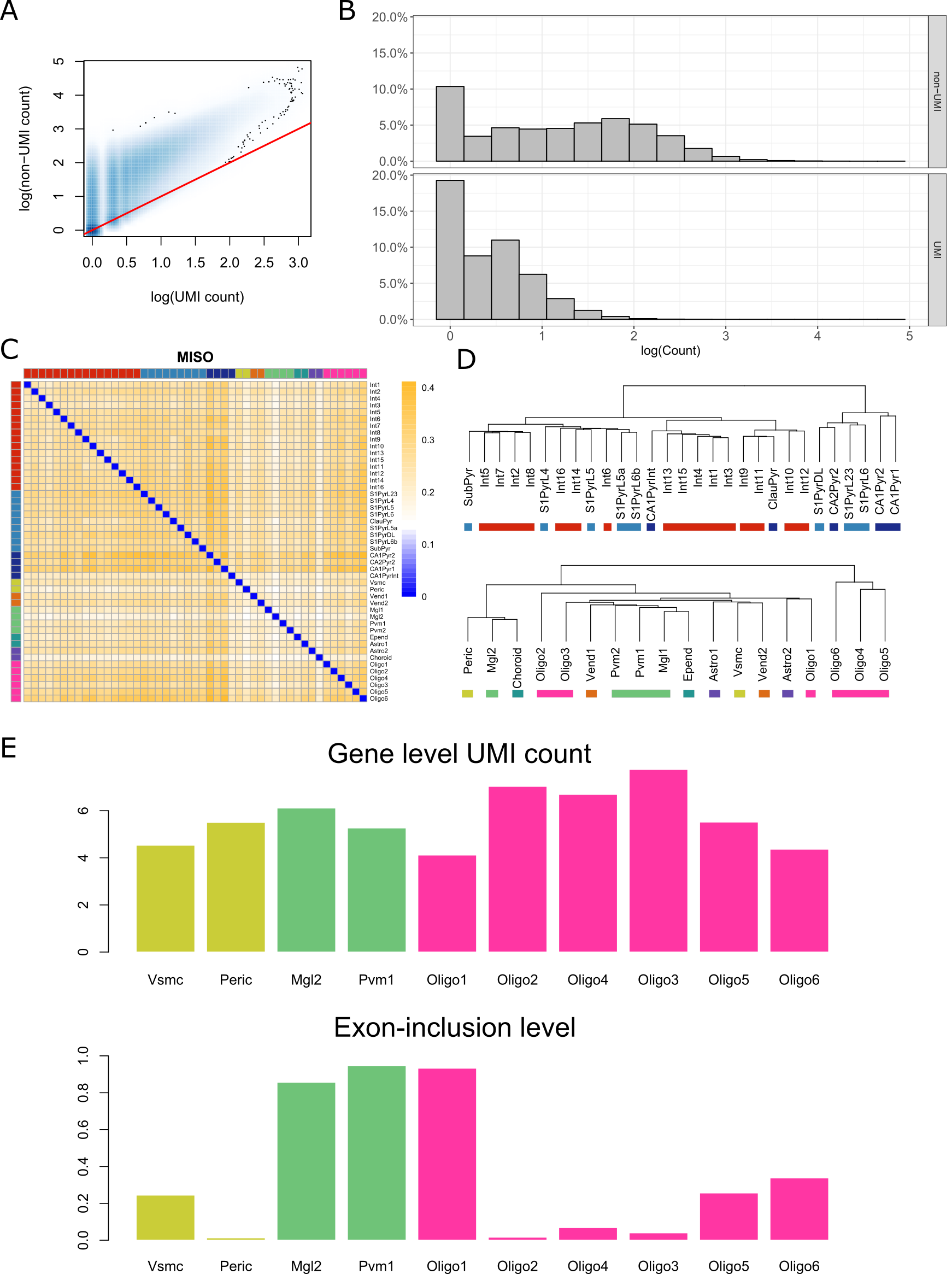

Supplement: S11 Fig — This dataset includes both UMI and non-UMI read counts. The UMI read counts were obtained by consolidating read counts for reads that originate from the same molecule based on UMI barcodes. This dataset allows us to compare UMI and non-UMI read counts systematically for DAS analysis. (A,B) Comparison of UMI and non-UMI read counts. (A) Scatter plot of UMI splicing informative read counts against non-UMI splicing informative read counts in log scale. Each point was summarized based on the same exon group in the same cell. Red dashed line represents identical line. (B) Distribution comparison between UMI and non-UMI splicing informative read counts. UMI counts are much smaller than non-UMI counts, suggesting that splicing analysis using UMI counts is more challenging. (C,D) Pairwise DAS comparison across nine major cell types from mouse cortex and hippocampus using MISO. Colors indicate nine major cell classes. (C) Heatmaps showing the proportion of detected DAS exon groups for each pairwise comparison between sub-cell types. (D) Dendrogram depicting cell classification results of the 47 sub-cell types. The distance metric between two sub-cell types is the proportion of detected DAS exons among all analyzed exons by MISO. MISO showed the worst performance in cell type classification as compared to SCATS, Census and DEXSeq in Fig 3. (E) Gene-level UMI counts and exon-inclusion level estimates of the flip exon from gene Gria1 across sub-cell types. Splicing quantification offers higher resolution of cellular heterogeneity than total gene expression. (TIF) [file pcbi.1007925.s011.tif]

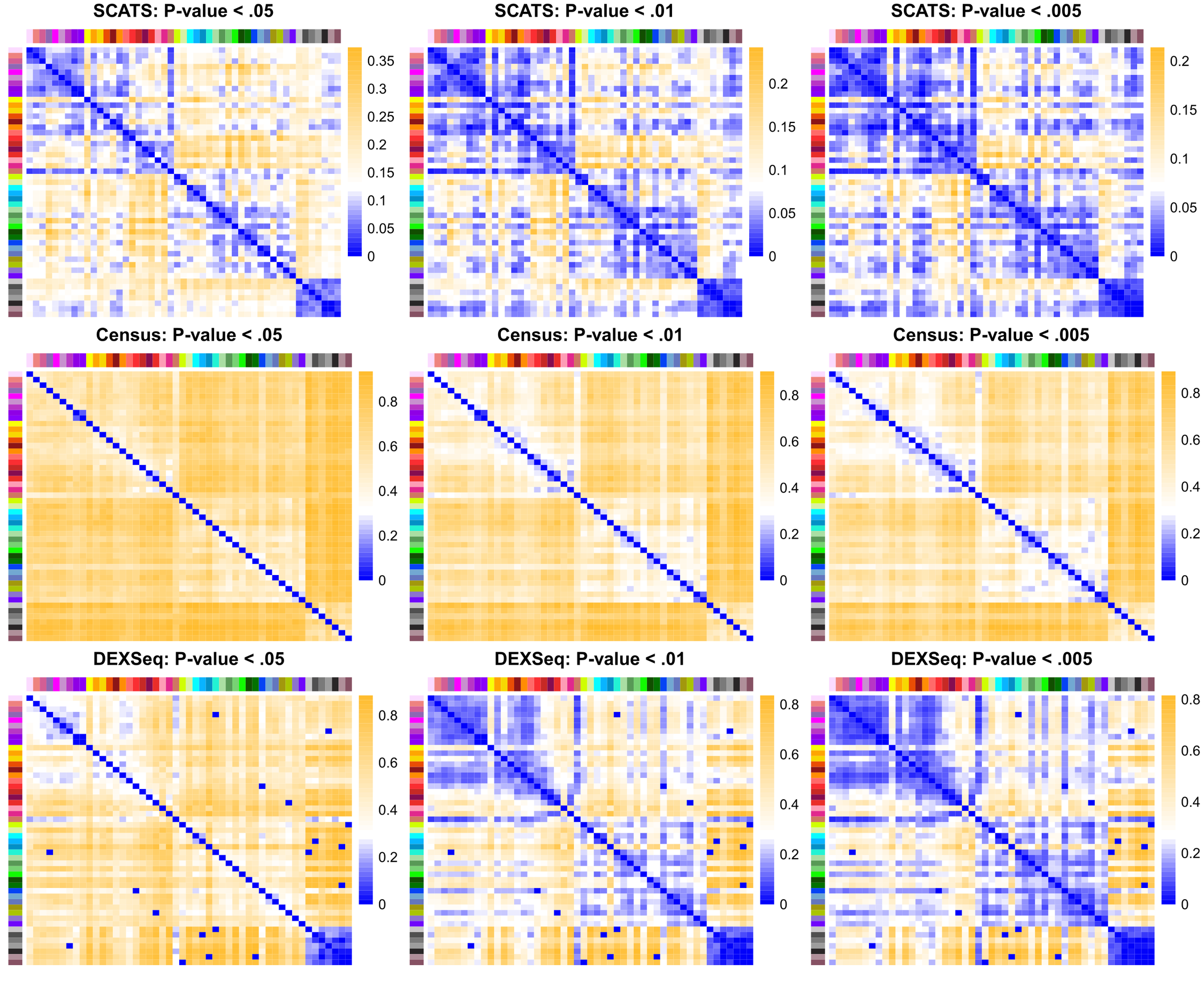

Supplement: S12 Fig — The analysis was conducted on 296 genes detected as the most differentially spliced genes by Tasic et al. based on MISO analysis. Heatmaps showing the proportions of detected DAS events for each pairwise comparison between 49 sub-cell types. The performance of SCATS, Census and DEXSeq was evaluated using different significance levels (α = 0.05, 0.01, 0.005). For within major cell class comparison (GABAergic, Glutamatergic, Non-neuronal), Census yielded much higher DAS detection rate (~0.6) than SCATS (<0.1), indicating possible inflated false positive results for Census as we expect to see smaller splicing differences for cells within the same major cell class. (TIF) [file pcbi.1007925.s012.tif]

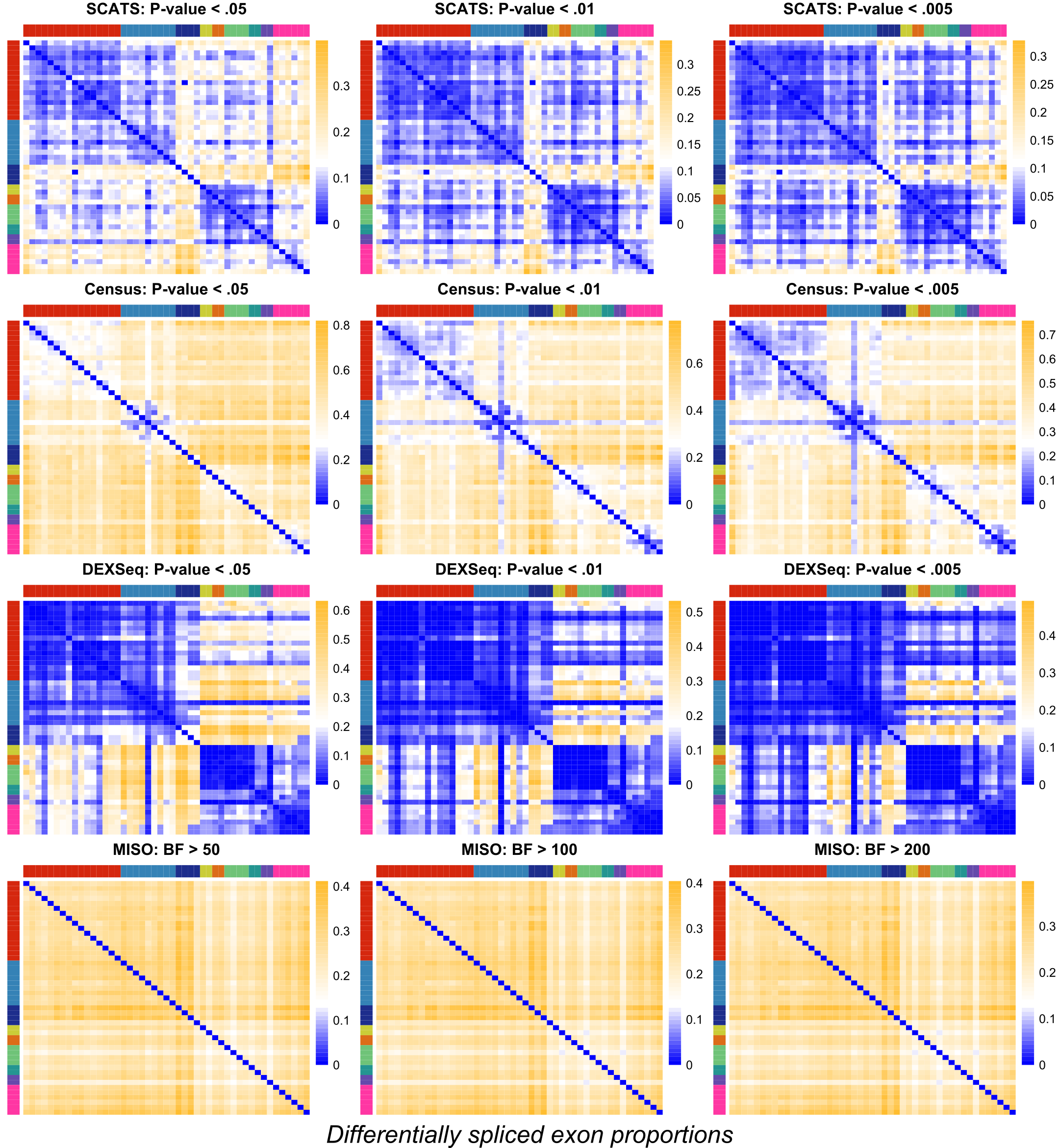

Supplement: S13 Fig — Heatmaps showing the proportion of detected DAS events (3,542 exon groups from 1,826 genes) for each pairwise comparison between nine major cell classes. The performance of SCATS, Census, DEXSeq and MISO was evaluated at different significance levels (α = 0.05, 0.01, 0.005 for SCATS, Census and DEXSeq, or Bayes factor: 50, 100, 200 for MISO). (TIF) [file pcbi.1007925.s013.tif]
